# Estimating the infant mortality rate from DHS birth histories in the presence of age heaping

**DOI:** 10.1371/journal.pone.0259304

**Published:** 2021-11-03

**Authors:** Julio Romero Prieto, Andrea Verhulst, Michel Guillot

**Affiliations:** 1 Department of Population Health, London School of Hygiene and Tropical Medicine, London, United Kingdom; 2 Population Studies Center, University of Pennsylvania, Philadelphia, PA, United States of America; 3 French Institute for Demographic Studies (INED), Paris, France; University of Salamanca, SPAIN

## Abstract

**Background:**

The infant mortality rate (IMR) is a critical indicator of population health, but its measurement is subject to response bias in countries without complete vital registration systems who rely instead on birth histories collected via sample surveys. One of the most salient bias is the fact that child deaths in these birth histories tend to be reported with a large amount of heaping at age 12 months. Because of this issue, analysts and international agencies do not directly use IMR estimates based on surveys such as Demographic and Health Surveys (DHS); they rely instead on mortality models such as model life tables. The use of model life tables in this context, however, is arbitrary, and the extent to which this approach appropriately addresses bias in DHS-based IMR estimates remains unclear. This hinders our ability to monitor IMR levels and trends in low-and middle-income countries. The objective of this study is to evaluate age heaping bias in DHS-based IMR estimates and propose an improved method for adjusting this bias.

**Methods and findings:**

Our method relies on a recently-developed log-quadratic model that can predict age-specific mortality by detailed age between 0 and 5. The model’s coefficients were derived from a newly constituted database, the Under-5 Mortality Database (U5MD), that represents the mortality experience of countries with high-quality vital registration data. We applied this model to 204 DHS surveys, and compared unadjusted IMR values to IMR values adjusted with the log-quadratic model as well as with the classic model life table approach. Results show that contrary to existing knowledge, age heaping at age 12 months rarely generates a large amount of bias in IMR estimates. In most cases, the unadjusted IMR values were not deviating by more than +/- 5% from the adjusted values. The model life table approach, by contrast, introduced an unwarranted, downward bias in adjusted IMR values. We also found that two regions, Sub-Saharan Africa and South Asia, present age patterns of under-5 mortality that strongly depart from the experience represented in the U5MD. For these countries, neither the existing model life tables nor the log-quadratic model can produce empirically-supported IMR adjustments.

**Conclusions:**

Age heaping at age 12 months produces a smaller amount of bias in DHS-based IMR estimates than previously thought. If a large amount of age heaping is present in a survey, the log-quadratic model allows users to evaluate, and whenever necessary, adjust IMR estimates in a way that is more informed by the local mortality pattern than existing approaches. Future research should be devoted to understanding why Sub-Saharan African and South Asian countries have such distinct age patterns of under-five mortality.

## Introduction

The infant mortality rate, commonly abbreviated IMR or _1_*q*_0_, is the probability of dying between birth and age of one year. As a key index of the social progress and health status of a population, the IMR is routinely monitored worldwide along with other mortality indicators. It is one of the key indicators tracked annually by the United Nations Inter-agency Group for Child Mortality Estimation (UN IGME), a group in charge of monitoring child mortality for the UN Millennium and Sustainable Development Goals. In many countries, however, vital registration systems –the gold-standard source of mortality information– are not reliable enough to provide precise estimates of the IMR. This is particularly true in low-and middle-income countries where vital registration systems often suffer from substandard coverage of deaths. In the last update of 2017, the UN Statistics Division estimated that less than 45% of countries benefited from a coverage of deaths that was above 90% [1]. In Sub-Saharan Africa, this was the case for only about 5% of countries.

Due to this lack of reliable vital registration systems, the estimation of mortality in low-and middle-income countries has depended largely on alternative sources of data such as nationally representative surveys. The Demographic and Health Surveys (DHS) Program, in particular, has constituted one of the most important sources of mortality information when no complete vital registration (VR) system is available. Since the 1980s, the DHS Program has collected and disseminated data on health and population in low-and middle-income countries. The full birth histories collected in these surveys have made it possible to monitor child mortality at relatively regular intervals. However, IMR estimates derived from these surveys have been regarded with caution. Preeminently, the UN IGME does not use direct estimates of the IMR based on DHS surveys [2]. This is despite the fact that they routinely use these surveys for monitoring the neonatal mortality rate as well as the under-5 morality rate (U5MR). The main concern associated with the IMR is the presence of heaping errors in the reporting of ages at death, in particular around age 12 months. As stated in the 2018 guide to DHS Statistics “heaping of deaths at age 12 months is common, and to the extent that it causes a transfer of deaths across the one-year boundary, infant mortality rates may be somewhat underestimated” [3]. However, the mechanism and the magnitude of this potential bias have not been systematically investigated.

Given the current state of knowledge, UN IGME prefers to rely on an indirect estimation procedure based on standard Model Life Tables (MLTs). MLTs provide equations predicting probabilities of dying at different ages for a given set of parameters related to the level and the age pattern of mortality. In this regard, MLTs take advantage of the high correlation found in human populations between death rates at different ages. The most common model is the one developed by Coale and Demeny [4] based on a set of historical VR data from Western countries. This model can predict, among other things, the IMR for a given level of the U5MR and a predefined age pattern of mortality. This approach has been implemented by the UN IGME for the estimation of the IMR in countries with defective VR data, taking the U5MR computed from difference sources –including DHS surveys– as a predictor.

However, in this approach, the choice of a predefined age pattern of mortality is an important limitation. In the Coale and Demeny version, the analyst has to choose between four patterns or *model families* (East, West, North, or South) resulting from the life tables of different country-years with similar attributes, irrespective of the level of mortality. In a more recent update, the UN [5] expanded the scope of MLTs to five additional families: Chilean, Latin American, Far Eastern, General, and South Asian. Nevertheless, two problems arise from the choice of family, in particular when predicting the IMR from the U5MR. First, the analyst has to rely on the observed relationship between mortality at ages 0–1 and 1–4 to establish the model age pattern. These two age groups define the only age breakdown available under age 5 in the MLTs. Hence, in order to predict the IMR based on the U5MR, the analyst must first observe their relationship in an alternative source of information. Most of the time, this alternative source is a DHS survey. Therefore, the analyst often ends up relying on an IMR estimate that is itself potentially biased due to age heaping. In addition, it is generally assumed that the choice of model does not change over time for a given country. This is problematic, because the age pattern of under-5 mortality can in fact change quickly over time [6]. Another problem stems from the discrete nature of the MLTs –there are nine model families only– while the range of possible values of the IMR for a given level of U5MR is in fact continuous. The implication is that MLTs cannot account for age patterns that are situated in-between the nine models.

In this paper, we propose to assess the quality of DHS-based IMR estimates by using a new model of under-5 mortality that overcomes the shortcomings of the MLTs. The model, whose details are provided elsewhere [7], builds on the log-quad approach initially proposed by Wilmoth et al. [8] and benefits form the newly constituted Under-5 Mortality Database (U5MD) –a data source that includes 22 detailed age groups from 0 to 5 years. Thanks to the granularity of the age breakdown, our assessment has one fundamental change when compared to the MLT approach. The choice of the age pattern of mortality can be directly informed by the data under study rather than by an alternative source of information that could be itself potentially biased by age heaping. Indeed, given the multiplicity of age groups in the model, robust/problematic data points can be selected/discarded around the month 12, as we propose in this paper. Thus, the model of under-5 mortality is fitted to DHS surveys to evaluate and potentially correct for age heaping at age 12 months. Our results show that the direct estimates of the IMR using DHS birth histories are more robust than previously believed. Therefore, this study brings a new, more positive view about the capacity of the DHS survey to correctly capture IMR levels. In addition, as a by-product, the results also provide new insights about the age patterns of under-5 mortality in developing countries.

## Materials and methods

### Data and data quality issues

#### DHS surveys used in the study

We used publicly-available standard DHS surveys with several exceptions. Since the focus of the paper is on age heaping around 12 months rather than other issues with the DHS surveys, we removed surveys for which the estimated level of U5MR is clearly biased. First, we discarded all the surveys whose data quality was shown to be insufficient through comparison with other sources of information. These surveys are those that were not retained by the expert panel of the UN IGME for monitoring the U5MR. Generally, these surveys were affected by strong underestimation biases potentially caused by omission of deaths or sampling errors. This selection does not imply that all retained surveys are completely free of bias in the level of U5MR, but at least it eliminates the most extreme cases. Second, we also discarded the surveys that suffered from the selection bias associated with HIV/AIDS. It is well known that the risk of dying among children whose mother deceased from HIV/AIDS is higher than average [9]. This generates a bias, because these higher-risk children are not captured in DHS surveys due to the fact that birth histories are collected among surviving mothers only. For that reason, we removed all the DHS surveys that were adjusted for that bias in the UN IGME database. As a result of this selection, we carried out the analysis on 204 surveys (see the list in the S1 Appendix). 88% of the discarded surveys (71 out of 81) were collected in the Sub-Saharan region.

#### Mortality estimation

In DHS surveys, women of reproductive age are asked to provide the date of birth and the age at death for all their children ever born. Dates of birth are reported by calendar years and months. Ages at death are reported in days if the death occurred during the first 30–31 days of life, in months for deaths up to 2 years of age, and in years up to age 5. Given this information, direct estimates of mortality can be computed for detailed age groups with a standard event/exposure procedure [10]. That is, period-and age-specific rates were estimated for both sexes combined by dividing the number of deaths by the number of person-years within each age group. We estimated these death rates for the 22 age groups that we use in the model. We computed the rates over a period of 10 years prior to survey in order to address potential transferences of births. Such type of error is generally attributed to interviewers. Typically, to avoid a supplementary module of questions about recent births, interviewers might displace (or omit) births beyond the cut-off date defined for that purpose (around 5 years prior to survey). Several researchers considered that such omissions and transferences of birth are more frequent among dead children, causing the systematic underestimation of recent estimates [10–13]. Finally, we transformed the age-specific rates into cumulative probabilities of dying under the assumption of a constant force of mortality within each of the 22 narrow age intervals.

#### Age heaping on the 12th month

**Fig 1:**
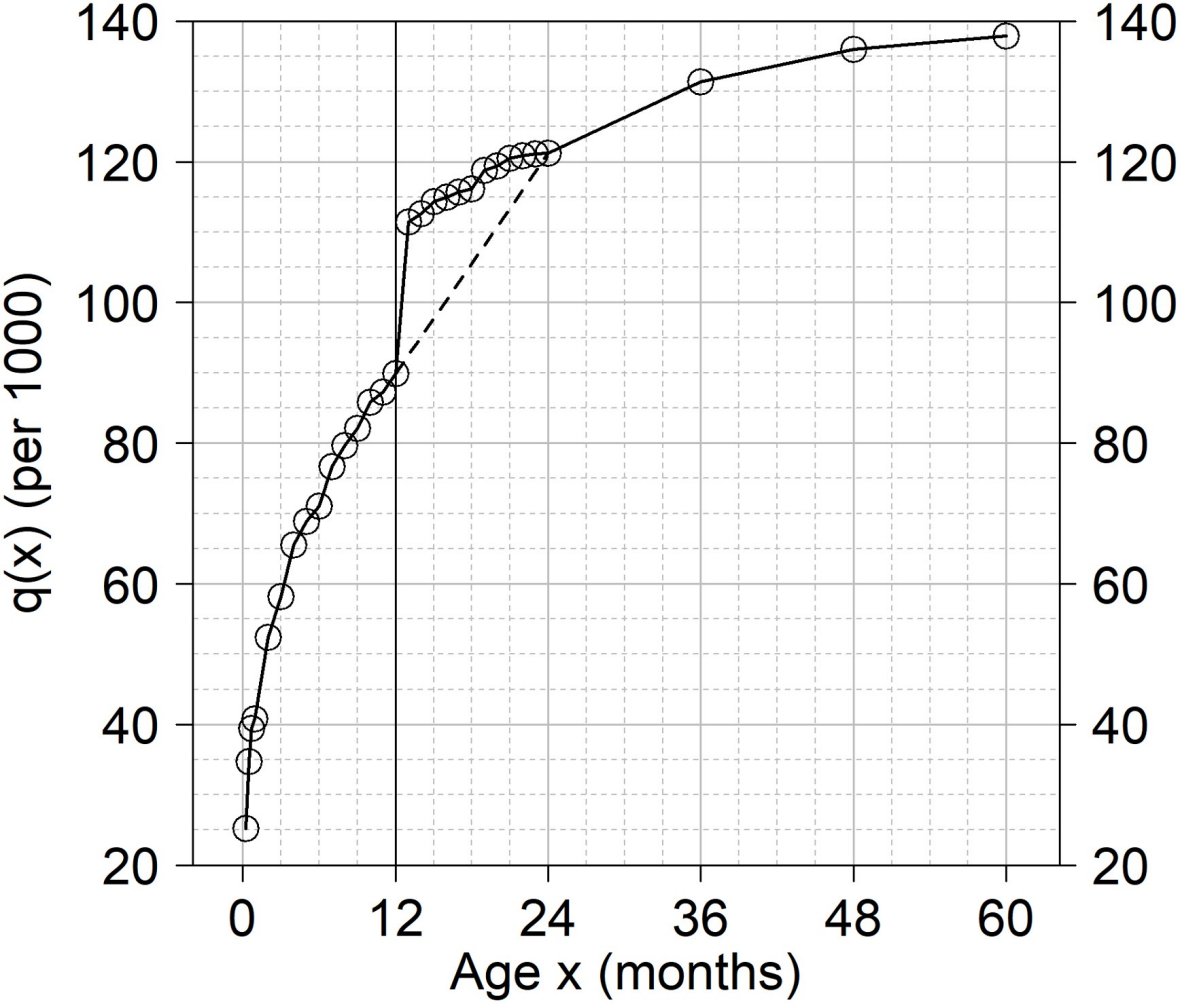
Percentage of under-5 deaths at ages 1 to 23 months in DHS surveys. Percentage estimated for each of the 204 selected surveys. Neonatal deaths not represented in the graph.

**Fig 2:**
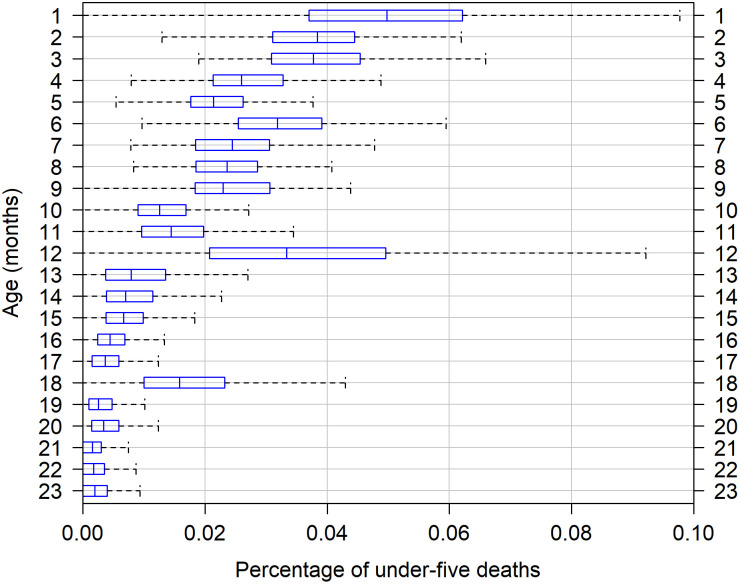
Cumulative probability of dying from birth to age *x* for the Bolivia 1989 DHS.

This paper is motivated by the existence in the DHS of heaping at age 12 months in reported ages at death, thus questioning the quality of DHS-based direct estimates of IMR. Fig 1 illustrates that phenomenon by showing the percentage of under-5 deaths at ages 1 to 23 months across the DHS surveys used in this study. The distribution shows that the proportion of deaths at age 12 months is considerably higher than the proportion of deaths at the adjacent ages. Heaping also occurs at ages 3, 6, and 18 months, although with a smaller magnitude. However, ages 3, 6 and 18 do not represent cut-off points for key indicators of mortality, so heaping at these ages is less problematic. As shown in Fig 2, age heaping at age 12 months can strongly distort the curve for the cumulative probabilities of dying from birth to age *x*, i.e., *q*(*x*). In some surveys, this is particularly evident for the estimates of *q*(*x*) by month of age; specifically, after the age 12 months. When age details between 13 and 24 months are not provided (dashed line), the distortion of the curve is not so clearly visible –but that does not mean that the potential bias is not present. Henceforth, we display the observed estimates by month of age for a better visualization of the issue.

Concerns about the potential underestimation of the IMR arises from the possible existence of an upward bias in reported ages at death. From this perspective, some deaths that occurred below the age of 12 months are misreported as occurring at the age of 12 completed months, i.e., above the threshold for the calculation of the IMR. Such upward age transfers thus erroneously exclude deaths from IMR calculations, generating a downward bias in the IMR. However, if heaping at age 12 months emerges from downward transfers, that is, if some deaths that occurred above 12 months are reported as occurring at the exact age of 12 months, then the IMR would not be biased. However, the direction of the age transferences (upward *vs*. downward) is not clear a priori. In DHS surveys, age at death is collected by month for the first two years of age. Thus, heaping would arise when the respondent does not know the child’s age at death in month and cannot be more accurate that “one year”. The interviewer would then either enter “year, 1” or “month, 12” (the latter being preferred). The answer of the respondent could also be in months but with some approximation, say 10–13 months. In that case, the interviewer would potentially enter “month, 12” as an estimation. For both the approximations in months and years, the transfer of deaths can potentially go upwards or downwards.

### Predicting and correcting the infant mortality rate

#### k-model

This paper relies on a recently-developed log-quadratic model of under-five mortality [7] in which the cumulative probability of dying between birth and age *x*, *q*(*x*), is assumed to be a log-quadratic function that depends on two entry values: i) the U5MR, here denoted *q*(5*y*); and ii) a parameter *k*, which determines the age pattern of mortality:

$$\begin{array}{c} \text{ln}\left[\text{q}\left(\text{x}\right)\right]=a_{x}+b_{x}\cdot ln\left[q\left(5y\right)\right]+c_{x}\cdot ln{\left[q\left(5y\right)\right]}^{2}+v_{x}\cdot k \end{array}$$ (1)

Empirically, the parameter *k* takes a continuous range of values between -1.1 and 1.5, thus replacing the choice among the nine model families of the MLTs. This is the reason that we refer to the model as the *k-model*. When *k* is equal to zero, the model predicts a full series of *q*(*x*) values from birth to age 5, corresponding to the model’s average age pattern of mortality. Positive values of *k* reflect a later age pattern of mortality captured by the model, that is with a higher-than-average concentration of deaths at older ages given a particular level of U5MR. Conversely, negative values of *k* correspond to an earlier age pattern, with a higher-than-average concentration of deaths at younger ages. The advantage of this model is the possibility to adjust the value of *k* by fitting (at least) any two probability probabilities of dying as far as their age boundaries correspond to one of the 22 exact-age cut-off points of the model between age 0 and 5.

The set of coefficients [a_*x*_, b_*x*_, c_*x*_, v_*x*_] were estimated on the basis of 1,219 country-years selected among the U5MD, providing high quality distributions of under-5 deaths by detailed age [7]. The database comprises VR data collected in industrialized, mostly Western, countries; and the covered period spans from the middle of the 19th century to the most recent years (up to 2016). All the country-years included in the database can be found in the Human Mortality Database [14], although the latter does not provide detailed age breakdown between 0 and 5.

**Fig 3:**
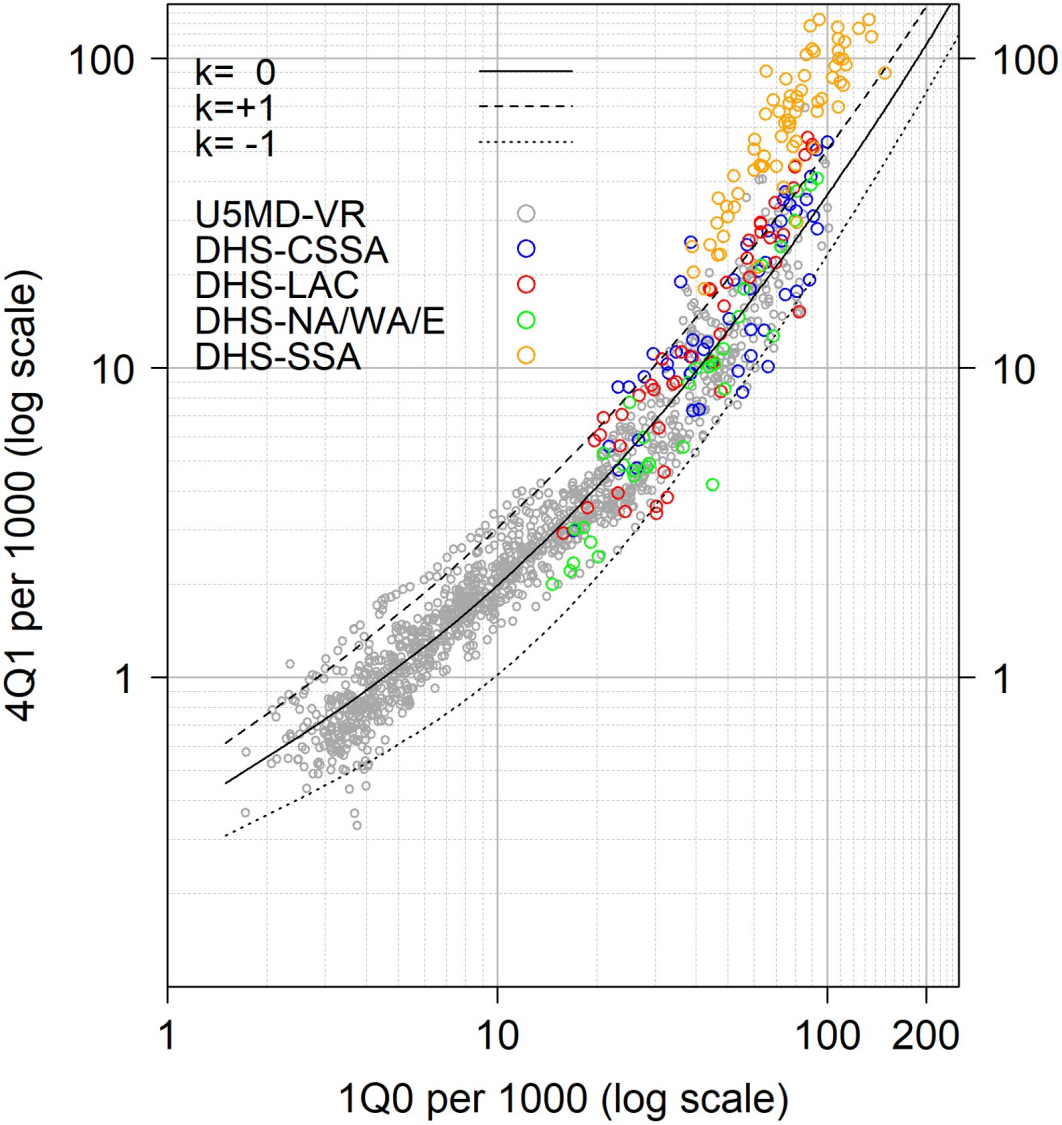
Data versus model predictions in the relationship between infant and child mortality. U5MD-VR is for Under-5 Mortality Database vital records. For the regional classification of DHS, CSSA is for Central, South, and Southeast Asia, LAC is for Latin America & Caribbean, NA/WA/E is for North Africa/West Asia/Europe, SSA is for Sub-Saharan Africa.

Being based primarily on to the Western experience, we do not expect the k-model to represent all possible age patterns of under-5 mortality. Fig 3 compares the relationship between infant and child mortality, denoted 1q0 and 4q1, in the selected 1,219 country-years of the U5MD and the DHS Surveys under study. The gray dots represent the Western experience as depicted by the VR data, while the colored dots correspond to the DHS estimates. The black lines indicate the span of the k-model, representing different patterns of mortality. Whereas a majority of DHS estimates fit within the boundaries of the model, most Sub-Saharan surveys are out of limits inasmuch as these surveys display a higher level of 4q1 for a given level of 1q0. While such deviations can be due to data errors, including heaping on age 12 months, it is also likely to be the result of a different –late– age pattern of under-5 mortality that was not observed in the Western countries. This later age pattern is likely associated with infectious and parasitic diseases, that have generally maintained mortality –after age 6 months– at a higher level than the observed in the West at similar levels of infant mortality [6].

#### Overview of the fitting method

We summarize our fitting procedure as follows: First, we estimated the optimal value of *k* –that is, the parameter that summarizes a population’s age pattern of under-5 mortality– for each DHS survey by fitting the observed cumulative probabilities of dying. The fit was carried out using ages that we considered not affected by age heaping at age 12 months, that is, ages less than or equal to 8 months and greater than or equal to 24 months. Second, we defined fitting criteria to determine if the model could correctly capture the age pattern of under-5 mortality observed in each survey.

The graphical and numerical outcome for each of the 204 DHS surveys used in this study is available in S1 and S2 Appendices respectively.

#### Determining the age pattern of under-5 mortality (estimating the optimal value of *k*)

Using Eq (1), we estimated the optimal value of *k* for each DHS survey by minimizing the weighted Root Mean Square Error of prediction at a given level of U5MR. The weight for each predicted probability was determined by the length of the preceding age interval. As in the case of the MLT approach to predicting IMR, the observed U5MR is assumed unbiased. To evaluate the impact of reporting errors around age 12 months, the estimation of *k* was performed after excluding the observed values of *q*(*x*) between 8 and 24 months, i.e., probabilities of dying located on either side of the IMR indicated by *q*(12*m*). This omission of data is justified by the need to minimize the potential impact of heaping on observed data. Particularly, we excluded the second year of age due to the way data are coded in DHS surveys. When the month of death is missing for the second year of age, we expect the age at death to be coded “Month, 12”. Therefore, such age misreporting can potentially affect the entire year interval. This omission of observed data is also justified by the fact that the *q*(*x*) information for the remaining age groups is highly efficient to infer the value of *k* for a given population. To prove that assertion, we compared the values of *k* estimated for each survey using *q*(*x*) values for all ages *vs*. after excluding ages 8 to 24 months.

The result of that test indicates that, on average, the absolute variation of *k* is only 0.1 with a standard deviation of 0.16. The relative variation in the prediction of the IMR is on average less than 1% with a standard deviation of less than 1.5%. Note that if the lower age bound is reduced from 8 to 6 months –allowing for more potential downward transfers– then the predicted IMR would have an average variation of less than 0.5% (with also less than 0.5% in the standard deviation of the variation).

#### Selecting surveys with good fit

Given the estimated value of *k*, two elements can potentially prevent the model from correctly capturing the age pattern of under-5 mortality in DHS surveys. First, the geographical and historical scope of the k-model is restricted to Western countries. Therefore, some age patterns that are specific to non-Western countries are not expected to be properly reproduced by the model. Second, DHS estimates are potentially affected by biases and errors specific to retrospective and survey data, such as omission of deaths or sampling errors. Consequently, the age pattern of mortality observed in a given DHS survey might be distorted and thus not well captured by the model.

To ensure we produced robust results, we discarded the surveys that are potentially affected by at least one of these two issues. To do so, we selected only the surveys that benefited from a close fit when applying the k-model, after establishing fitting rules based on the mean relative error of predictions. In order to avoid the potential imbalance in the fitting before 8 months and after 24 months, we established maximum error values from 0 to 8 months and between 24 and 60 months separately. We determined these maximum values from the distribution of the relative mean errors obtained on these two age ranges, when applying the k-model to the VR data that were used to estimate the coefficients [a_*x*_, b_*x*_, c_*x*_, v_*x*_]. This is based on the knowledge that the model fits the VR data extremely well [7]. As a rule, we took as maximum error values those corresponding to the 90% and the 99% best fits. These maximum values of error are given in Table 1. Note that the 90% criterion is more conservative. When the relative mean errors computed for a DHS survey were found between the boundaries given in Table 1 for both age ranges, we concluded that the age pattern of under-5 mortality was correctly captured by the model. Thus, we considered that in such cases a robust prediction of the IMR would be generated by the k-model. When this criterion was not satisfied, we excluded the survey from the analysis.

**Table 1:** Maximum values of mean error of predictions (%) defining a good fit of the k-model.

Age range	Precision criterion	
	90%	99%
0 to 8 months	[-1.34%, 1.14%]	[-2.50%, 1.96%]
24 to 60 months	[-1.46%, 1.60%]	[-2.16%, 3.20%]

These values were estimated with the Under-5 Mortality Database (U5MD) vital records from which the k-model is derived.

## Results

In Table 2, we indicated the number and percentage of surveys included in the analysis, i.e., the surveys that passed the fitting test. In total, the 90% criterion restricts the analysis to 61 DHS surveys (30%), while the 99% criterion allows us to work with 109 surveys (53%). The breakdown by world region shows that most of the excluded surveys were collected in Sub-Saharan Africa. Only one survey out of 73 (Ethiopia 2016) was kept under the looser fitting criterion (99%). This is consistent with the specificities found for this region compared to the VR data. In contrast, in the other regions, the model fitted a majority of surveys, in particular when considering the 99% fitting criterion. This is the case for the groups of countries in North Africa, West Asia and Europe (85% of surveys included) and Latin America and the Caribbean (85%). The group of Central, South, and Southeast Asian countries benefits from a slightly lesser proportion of good fit (78%). Results for individual surveys (see below) show that some Asian countries display similar age pattern of under-5 mortality than in Sub-Saharan Africa.

**Table 2:** Numbers and percentages of surveys included in the analysis according to the 90% and 99% maximum values of fitting error.

World Region	90%			99%		
	Total	Incl.	% Incl.	Total.	Incl	% Incl.
Central, South & Southeast Asia	51	21	41%	51	40	78%
Latin America & Caribbean	47	26	55%	47	40	85%
North Africa/West Asia/Europe	33	14	42%	33	28	85%
Sub-Saharan Africa	73	0	0%	73	1	1%
Total	204	61	30%	204	109	53%

The selected surveys represent a pool of surveys for which we are confident that the k-model can summarize the underlying age pattern of under-5-five mortality and thus produce a robust prediction of the IMR. On the basis of these surveys, we evaluated the magnitude of the bias affecting the observed IMR estimates. In addition, we compared our predictions with values predicted on the basis of the MLT approach. To produce these MLT estimates, we selected the model families established by the UN IGME for each country.

**Fig 4:**
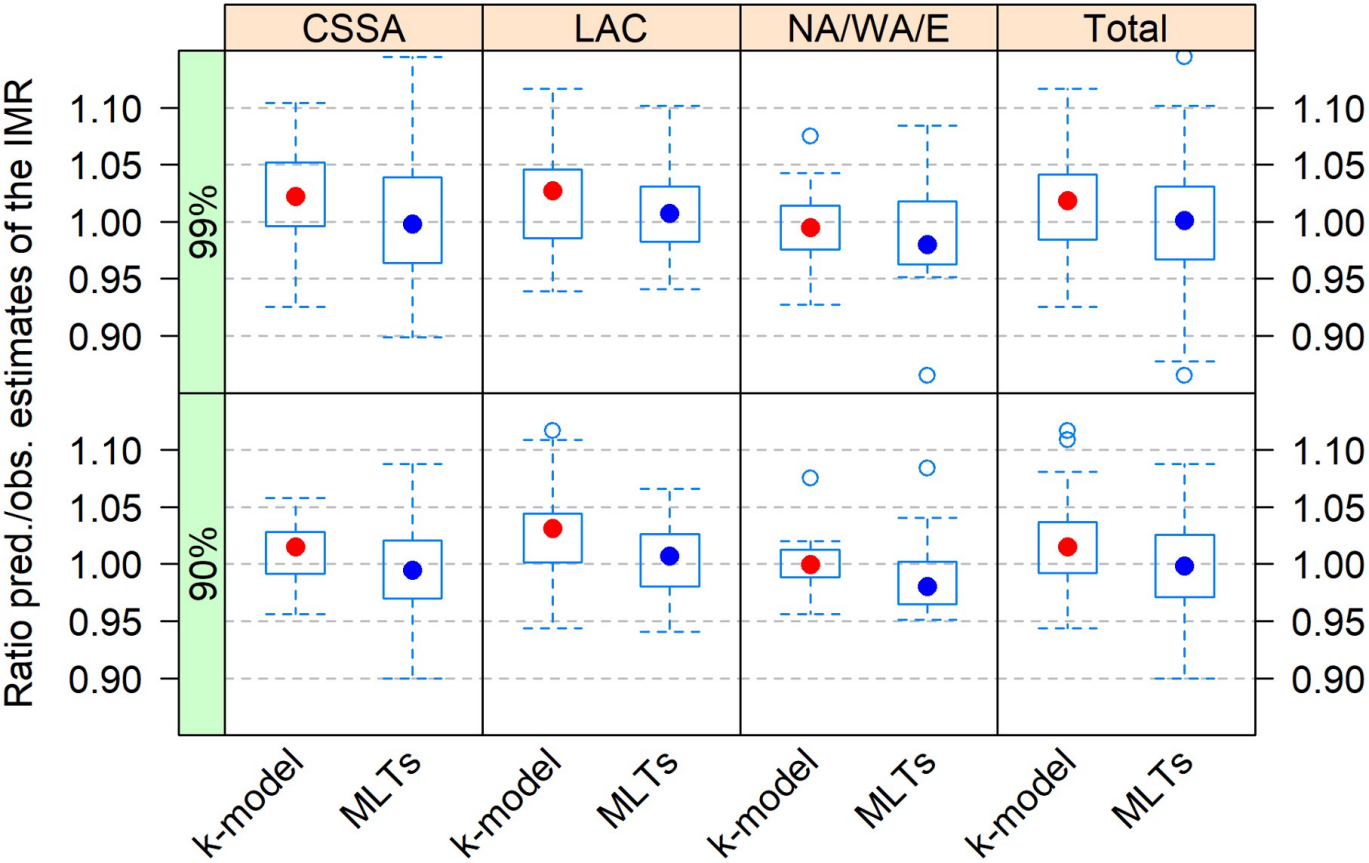
Predicted vs observed ratio of the IMR in individual DHS surveys by world region with predictions made using the k-model or MLTs. CSSA is for Central, South, and Southeast Asia. LAC is for Latin America & Caribbean. NA/WA/E is for North Africa/West Asia/Europe. Sub-Saharan Africa results are not showed because they include only one survey.

Fig 4 displays predicted *vs*. observed ratios of the IMR in retained DHS surveys, by world region. The results are similar for both the 90% and 99% maximum values of fitting errors: the k-model tends to predict values that are on average about 2% higher than the observed ones, suggesting that observed IMRs in the DHS tend to be underestimated. Although this result is consistent with expectations, the magnitude of the bias is small. In total, most ratios are situated between 0.95 and 1.05: 74% with the 99% fitting criterion and 87% with the stricter one. For the looser criterion, the ratio hardly goes beyond 1.10. In North Africa, West Asia, and Europe it does not go over 1.05.

**Fig 5:**
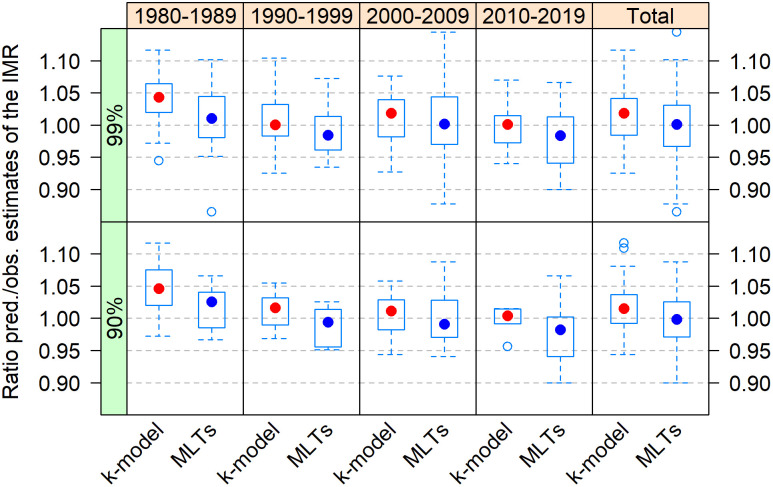
Predicted vs observed ratio of the IMR in individual DHS surveys by decade with predictions made using the k-model or MLTs.

Fig 5 displays the same results but classified by decade. It shows that the bias was larger during the first decade of the DHS Program and was reduced thereafter. This is likely the result of the early awareness of the issue and the subsequent improvement of the data collection during the fieldwork.

In contrast with the k-model, the traditional MLT approach predicts lower values of IMR, larger dispersion, and less bias on average. The MLT results also indicate that the IMR would tend to be more overestimated than underestimated in DHS surveys, especially for recent periods.

Overall, our results suggest that the direct estimates of the IMR using DHS birth histories are more robust than previously thought. This conclusion holds for the two criteria adopted to evaluate the goodness of fit. However, in order to use the k-model for correction, it is necessary to evaluate surveys individually, in particular those whose prediction errors are close to the boundaries of the fitting criteria. The analysis of individual surveys also permits to better understand the mechanisms producing the heaping at age 12 months and thus the bias in the IMR.

**Fig 6:**
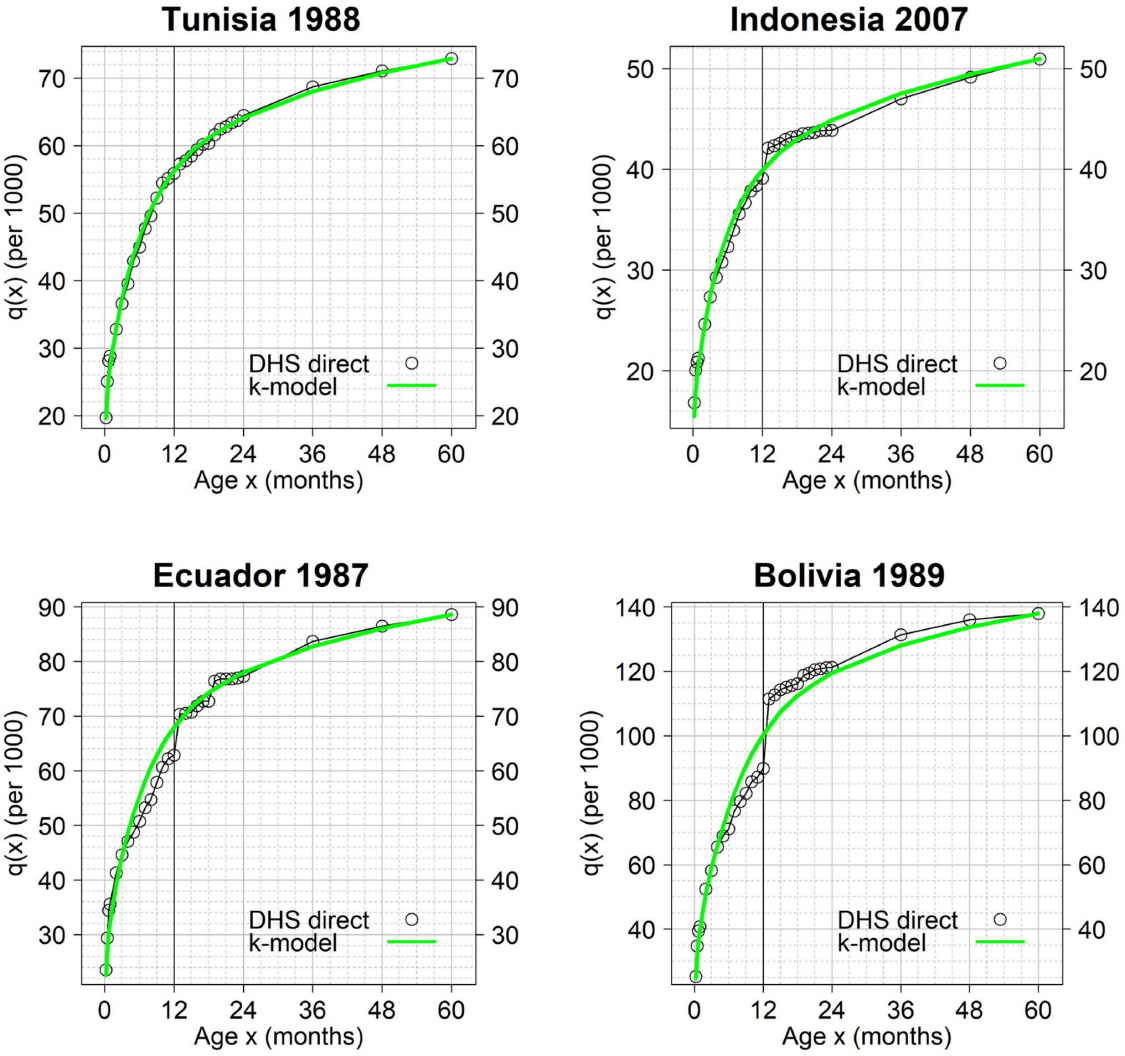
Examples of surveys with very good fit of the k-model. Prediction errors are within the 90% maximum values. The k-model was fitted to the observed *q*(*x*) values using ages 0–8 and 24–60 months only. The ticker vertical line indicates the comparison between the observed and predicted infant mortality rate (IMR).

**Fig 7:**
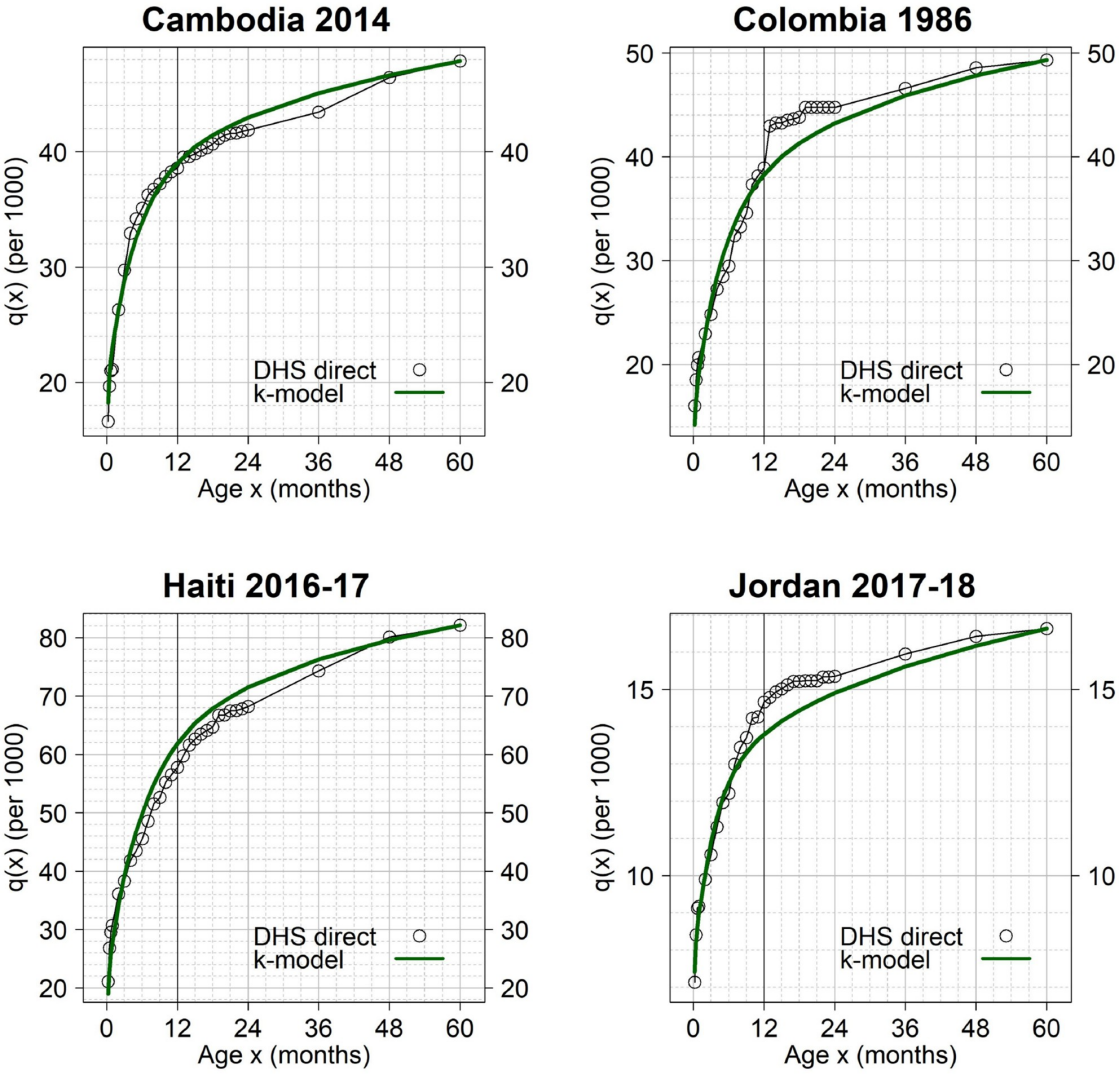
Examples of surveys with relatively good fit of the k-model. Prediction errors are between the 90% and 99% maximum values. The k-model was fitted to the observed *q*(*x*) values using ages 0–8 and 24–60 months only. The ticker vertical line indicates the comparison between the observed and predicted infant mortality rate (IMR).

**Fig 8:**
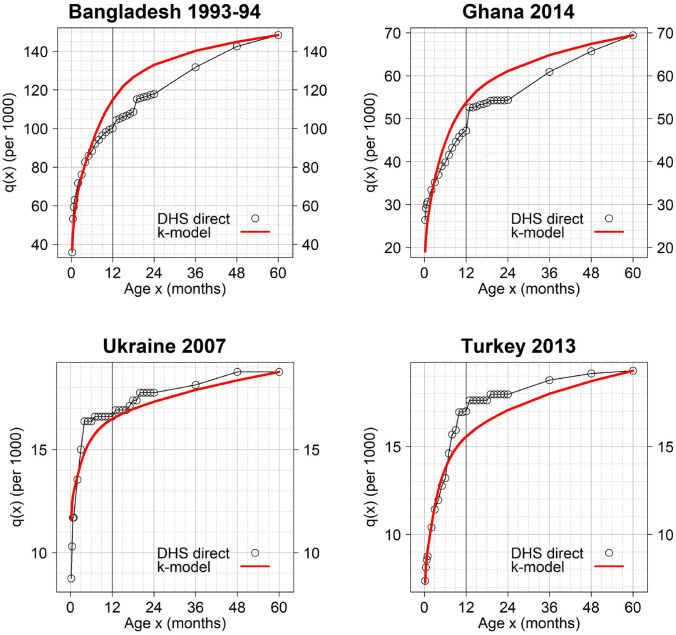
Examples of surveys excluded from analysis due to poor fit of the k-model. Prediction errors are outside the 99% maximum values. The k-model was fitted to the observed *q*(*x*) values using ages 0–8 and 24–60 months only. The ticker vertical line indicates the comparison between the observed and predicted infant mortality rate (IMR).

In the next three figures, we compared the observed and predicted cumulative probabilities of dying for selected surveys. Figs 6 and 7 show surveys satisfying the 90% and 99% maximum values of prediction error respectively, while Fig 8 shows excluded cases.

Therefore, the results presented in Fig 6 display some of the country-years that are best fitted by the k-model. These results exemplify the capacity of the k-model to capture age patterns of under-5 mortality experienced in a variety of Non-Western countries. Tunisia 1988 is a case where no issue in the measurement of the IMR is noticed, even though the data were collected in the early period of the DHS Program.

Indonesia 2007 (Fig 6) represents a pattern commonly found in the pool of selected surveys: some heaping at age 12 months is observed, but no impact on the mortality curve before one year is visible. Therefore, in these cases, all the misreported deaths must have been the result of downward transferences which have no effect on the IMR. These downward transferences of age correspond to children that actually died between 1 and 2 years old but whose mother could not provide a precise estimate of the age at death in months. The interviewer must then have assigned “Month, 12” to these children. This example illustrates why we found limited underestimation of the IMR at the global level: our results show that a large part of the heaping at age 12 months appears to result from downward transferences rather than upwards ones.

In contrast, Ecuador 1987 (Fig 6) gives an example where the misreporting of deaths was primarily the result of upward transferences, with some deaths in the later part of the first year of age being misreported as deaths at age 12 months. Bolivia 1989 (Fig 6) is seemingly the clearest example of the action of both effects, that is upward and downward transferences of age at the same time.

In Fig 7, Cambodia 2014 and Colombia 1986 show outcomes similar to Tunisia 1988 and Indonesia 2007, respectively. In the former case, there is no heaping; and in the latter, there is evidence of downward transference of deaths. In both cases, the fit looks excellent even if they meet the 99% criterion only. This shows that the criteria of inclusion can be somewhat arbitrary, thus the importance of analyzing each survey individually.

Note that the upward or downward corrections suggested by the k-model are not always related to age heaping. These corrections can also arise from the discrepancy between observed and predicted age patterns of mortality, even if the fit is relatively good; an outcome that is illustrated in Fig 7 by Haiti 2017–16 (predicted/observed IMR = 1.07) and Jordan 2017–18 (predicted/observed IMR = 0.94). These cases contribute to the dispersion of the global results but do not affect our main conclusion, i.e., that the impact of age heaping at age 12 months on IMR estimates in DHS surveys is limited –at least among surveys for which the k-model was able to capture the underlying age pattern of under-5 mortality.

In Fig 8, Ghana 2014 and Bangladesh 1993–94 are representative of patterns observed among excluded surveys. Typically, in the course of the first year of age the k-model starts producing a large overestimation of the level of mortality, missing thus what seems to be the late age pattern of under-5 mortality specific of that region. Indeed, both surveys show clearly that the lack of fit is distributed all over the curve. This type of discrepancy is observable for most Sub-Saharan surveys in the S2 Appendix. Age heaping at age 12 months could potentially be contributing to the poor fit. Other potential types of data error, such as death omissions at early ages, could also have affected the observed age pattern. However, the systematic pattern of discrepancy across countries supports the hypothesis of a specific –late– age pattern of under-5 mortality associated with infectious diseases.

Bangladesh provides a clear example of the existence of this age pattern in South Asia. In the 1970s, at the onset of the mortality transition there, a significant proportion of infant and child deaths was due to diarrheal diseases followed by acute respiratory infections. This kind of complications occurs after 6 months of age with the transition to solid diet and spread beyond infancy. However, over time, there has been an important shift from acute infectious diseases to chronic non-communicable diseases. Ahsan Karar et al. [15] showed that between 1986 and 2006 deaths due to infectious diseases, in particular diarrhea, dysentery, tuberculosis, and respiratory diseases, were reduced from 50% to 11% of all under-5 deaths. This transition can be observed across 7 DHS surveys collected between 1993 and 2014 (see the S1 Appendix). The improvement of the fit over time reflects the disappearance of this late age pattern of under-5 mortality specific to some epidemic contexts. This progressive disappearance together with the reduction of infectious diseases provide another clue that this age pattern is real and not a data artifact. We found a similar transition in other countries from Asia that benefited from a long series of surveys: Nepal and Philippine (see the S2 Appendix).

**Fig 9:**
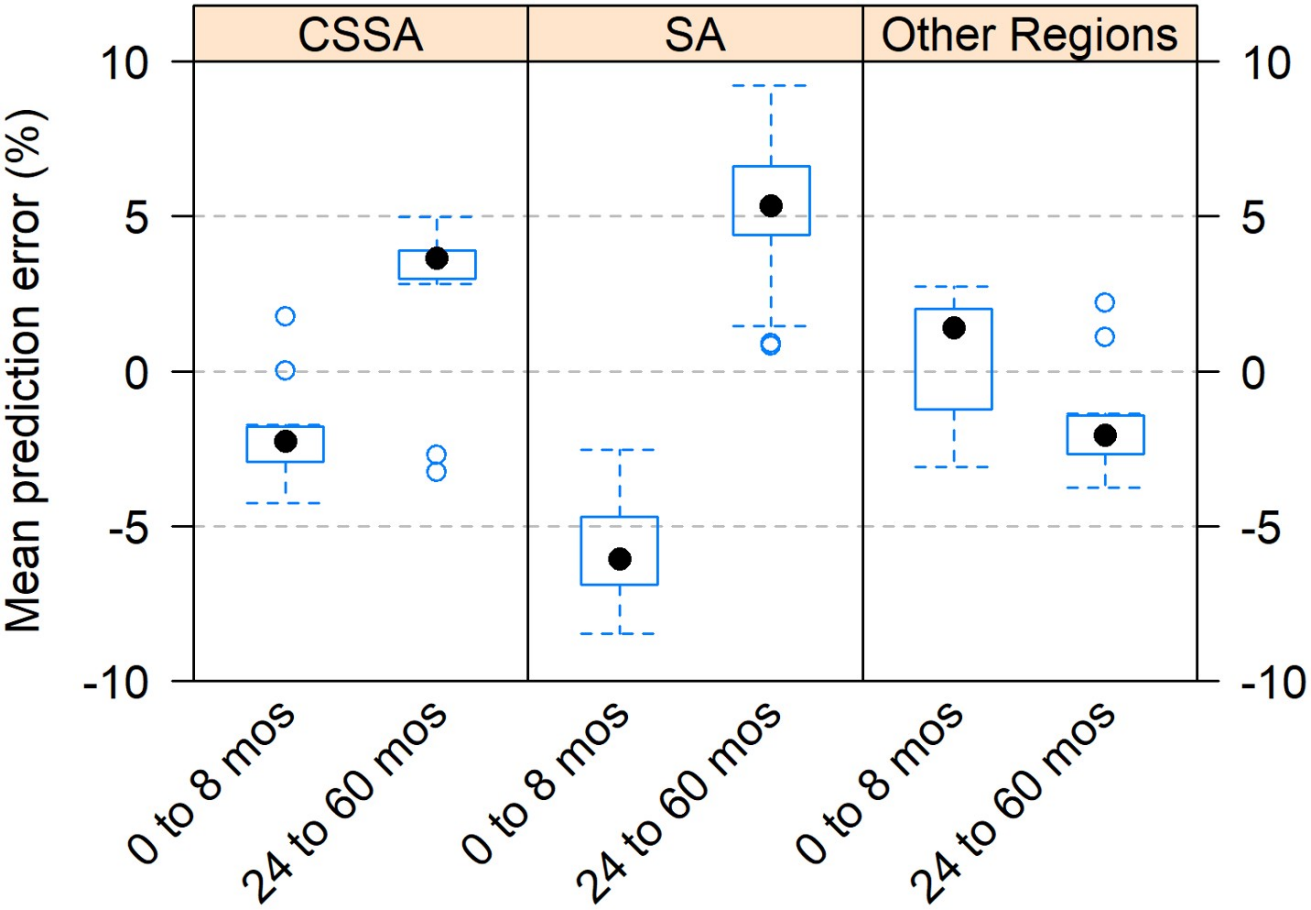
Mean prediction error (%) of k-model among excluded surveys by region. CSSA is for Central, South, and Southeast Asia. SA is for Sub-Saharan Africa.

More broadly, Fig 9 shows that in both Asia and Sub-Saharan Africa, the excluded surveys present an unusually late age pattern of under-5 mortality. Contrary to the other regions, the predictions of the k-model for the age above 24 months overestimate the observed values of *q*(*x*) in Asia and Sub-Saharan Africa. In addition, Fig 9 shows that the excluded surveys in Asia and Sub-Saharan Africa had a poor fit at younger ages as well. Indeed, we found that mortality are ages 0 to 8 months was systematically underestimated by the k-model. On average for both regions, that underestimation was -5.4%. Moreover, the discrepancy between observed and predicted *q*(*x*) increases up to -31.5% when focusing on the neonatal period (not shown). This result indicates another specificity of the age pattern of under-5 mortality in some Asian countries and most of Sub-Saharan Africa.

Fig 8 also shows results for Ukraine 2007 and Turkey 2013, i.e., two countries that are expected to be well fitted by the k-model due to their geographical proximity with the countries that were used to develop this model. However, both cases show how sizeable data errors can be due to low levels of mortality. At low levels of mortality, DHS surveys will have zero deaths in many of the narrow age groups, especially after 12 months, generating rather flat *q*(*x*) curves during the later portion of the 0–5 age range. In Ukraine, the age pattern is particularly irregular and could not be captured by the model. In the case of Turkey 2013, the pattern is less uneven but was also poorly fitted. Note that the earlier DHS surveys collected in Turkey display a fit that ranges from relatively good to very good (see the S2 Appendix). This difference among surveys of a same country confirms the impact of data artifacts arising from small sample size and low mortality levels. Here, the difference over time cannot be explained by a progressive modification of age pattern of mortality over time as in the case of Bangladesh discussed above. In fact, the group of countries including Turkey and Ukraine (North Africa, West Asia, and Europe) is the one for which our model performs best (Table 2). Note that these two DHS surveys (Ukraine 2007 and Turkey 2013) were the last surveys used by UN IGME to monitor under-5 mortality. Since 2007 and 2009 respectively, UN IGME has only relied on the VR data to monitor mortality in the two countries, which is the more adequate source of data when completeness is reached.

## Discussion

In this study, we examined the extent to which heaping of reported ages at death at 12 months may impact DHS-based IMR estimates. Because of this well-known problem, analysts have justifiably questioned the quality of the IMR values directly estimated from DHS surveys. It has been assumed that heaping at age 12 months would underestimate this important indicator of population health, but the magnitude of the bias had thus far not been thoroughly evaluated.

To contribute to the understanding of this issue, we proposed using a new model to evaluate the IMR estimated from 204 DHS surveys. We used this model to predict accurate estimates of the IMR based on the observed age pattern of mortality. To ensure that the age patterns were correctly captured, we used two fitting criteria. The conservative criterion retained 47% of non-Sub-Saharan surveys, while the more inclusive criterion kept 80% of these surveys (also excluding Sub-Saharan Africa). The aim of using two criteria was to demonstrate that the main finding of this study was not dependent on this choice.

The main finding of this methodological inquiry is that the underestimation of the IMR is small. We found that most of the observed values of the IMR were not deviating by more than +/- 5% from the predicted value. This suggests that heaping at age 12 months in reported ages at death arises to a large extent from downward transferences of age that do not impact mortality below 12 months. Therefore, this study brings a new, more positive view about the capacity of DHS surveys to correctly capture IMR levels.

This finding thus has important implications for monitoring infant mortality in low-and middle-income countries using DHS surveys. First, we recommend applying the k-model (or the results of the k-model available in S1 Appendix) as a starting point whenever age heaping is of concern in a given survey. The amount of difference between observed values of the IMR *vs*. values predicted by the k-model can then be used as a guide for deciding whether the IMR adjustment is necessary. Whenever the difference is less than 5%, we recommend using the unadjusted, direct estimates. Our results show that this is the most likely outcome. When the k-model produces an adjustment higher than 5%, however, we recommend using the corrected estimates after an individual examination of the model fit. Due to model’s lack of fit in Sub-Saharan African countries, these recommendations only apply to countries outside the Sub-Saharan Africa region.

Another implication of our study is that the MLT approach currently used by the UN IGME to estimate the IMR seems unwarranted. While some of the limitations of the MLT approach are similar to those of the k-model –limited geographical scope of the model, potential data errors, and assumption of unbiased U5MR (see below)– it relies on the choice of an age pattern that is not well informed empirically. This contrasts with our approach where the age pattern is directly informed by the data via the estimation of *k*. For that reason, we consider the results of our approach to be more robust. In contrast with results from the MLT approach, we do not find evidence that unadjusted IMR levels have a tendency to be too high due to age heaping. One area of agreement between the two approaches, however, is that the bias due to age heaping at age 12 months is rather limited.

Our study has some limitations. First, the proposed approach cannot be applied to all DHS surveys. Despite the good performance of the model in various world regions, we had to exclude almost all Sub-Saharan surveys. Indeed, in this region, due to a distinct age pattern of under-5 mortality, the model could not correctly fit the observed data. It is possible that the impact of age heaping may be different in these discarded surveys. It is also possible that the results would be similar due to the uniform methodology of DHS. To verify these possibilities, further investigation is needed.

However, meanwhile, we do not recommend the use of the classic MLT approach in Sub-Saharan Africa. Indeed, the geographical scope of the MLTs is similar to that of the VR data underlying the k-model, and thus suffer from the same limitation. Since the k-model does not fit the Sub-Saharan pattern, there is no reason to believe that the classic MLTs would provide a better basis for adjusting mortality in that region. Therefore, future work should expand the geographical and epidemiological scope in the modeling of the age patterns of under-5 mortality. Recently, [16] proposed to model the age distribution of under-5 deaths in Sub-Saharan Africa using the DHS surveys. This avenue is warranted, but we consider that further assessment of the data quality in DHS survey is required prior to modeling age patterns of under-5 mortality in low-and middle-income countries. This would require gathering high-quality information collected from sources other than DHS surveys, including demographic surveillance sites, sample and non-sample vital registration systems, etc. In the meantime, it might be preferable to keep the unadjusted values of the IMR rather than apply the MLT approach to Sub-Saharan surveys.

Our study provides insightful lessons toward this research avenue, in particular about the Sub-Saharan age pattern of under-5 mortality. The systematic character of the deviation between observed and predicted mortality estimates across ages indicated that the discrepancy was the result of an actual age pattern of under-5 mortality specific to that region. This upholds the hypothesis that Sub-Saharan Africa has experienced a late schedule of under-5 mortality –associated with infectious and parasitic diseases– that was not observed in Western history. We provided evidence that a similar age pattern also existed in some Asian countries. Specifically, we showed that in Bangladesh the disappearance of this specific late age pattern of under-5 mortality was concomitant with the reduction of infectious diseases.

In addition, we also found that this late age pattern was associated with a very early one, i.e., a high level of neonatal mortality given the level of under-5 mortality. To our knowledge, this specific deviation from the age pattern observed in Western countries at the early ages has never been shown before. Therefore, future work is needed to understand and capture this double deviation –early and late– in models of the under-5 mortality age pattern.

A second limitation is that data quality issues might also have affected our results. We showed that variations in the age pattern of mortality were not necessarily real but rather the outcome of errors in the estimates unrelated to age heaping, e.g., in the series of surveys collected in Turkey. It is not clear if these were sampling or non-sampling errors, but small fluctuation in *q*(*x*) may indeed produce a poor fit of the data. Nonetheless, in order to minimize this issue, we also showed that substantial modifications in the fitting rules did not impact substantially the overall evaluation of the IMR.

Finally, our analysis was based on the assumption that the level of the U5MR was unbiased. Although we excluded the DHS surveys with obvious data quality issues, we cannot guarantee that omissions of deaths have not biased our results to some extent. This sort of data errors is the most difficult to track. Some concerns touch upon the potential omissions of children who died very young, particularly as neonatal and early neonatal deaths [17]. Further work is needed to evaluate this issue. However, our results suggest that the confidence in DHS-based IMR estimates should not be notably lower than the confidence usually attributed to the U5MR.

Despite these limitations, our results show that, on the whole, age heaping around 12 months in DHS surveys does not appear to generate a strong bias in direct estimates of the IMR. We conclude that IMR estimates of directly estimated from DHS surveys are less affected by age heaping bias than previously thought.

## Supporting information

S1 Appendix List of used DHS surveys and numeric results. (PDF)

S2 Appendix Graphical results for individual DHS surveys. (PDF)

## Abbreviations

DHS: Demographic and Heath Surveys
IMR: infant mortality rate
MLTs: Model Life Tables
U5MD: Under-5 Mortality Database
U5MR: under-5 mortality rate
VRs: vital records
