# Supplementary material for: Estimating the infant mortality rate from DHS birth histories in the presence of age heaping
Source: PLoS One. 2021 Nov 3;16(11):e0259304. doi: 10.1371/journal.pone.0259304 (PMC8565760; doi:10.1371/journal.pone.0259304)
Supplement: S1 Appendix — (PDF) [file pone.0259304.s001.pdf]

S1 Appendix: List of used DHS surveys and numeric results

This appendix provides for each survey:

- The observed value of the under-five mortality rate (U5MR)
- The estimated optimal value of k for included surveys
- The mean prediction error for the age ranges 0-8 months and 24-60 months
- The inclusion/exclusion outcome given the fitting criterion
- The observed and estimated values of the infant mortality rates (IMR) as well as their ratios

	Survey	U5MR	k	Mean Prediction Error (%)		Selection	IMR			Ratio	
				0 to 8 mos	24 to 60 mos		Obs. (1)	k-Model (2)	MLT (3)	(2)/(1)	(3)/(1)
1	Albania 2008-09	21.87	0.29	0.89	−2.14	Included (99%)	19.19	17.79	19.50	0.93	1.02
2	Angola 2015-16	79.33	1.09	−4.36	4.88	Excuded	49.97	56.91	52.81	1.14	1.06
3	Armenia 2000	48.80	−0.66	1.99	−1.84	Excuded	44.79	41.83	41.71	0.93	0.93
4	Armenia 2005	33.47	−0.19	0.05	−0.53	Included (90%)	27.67	28.02	30.00	1.01	1.08
5	Armenia 2010	21.20	0.49	1.91	−3.75	Excuded	18.20	16.95	18.91	0.93	1.04
6	Azerbaijan 2006	56.95	−0.23	0.02	−2.02	Included (99%)	48.80	46.68	48.01	0.96	0.98
7	Bangladesh 1993-94	148.44	−0.44	−2.53	4.98	Excuded	100.15	114.89	102.52	1.15	1.02
8	Bangladesh 1996-97	126.99	−0.29	−2.28	3.71	Excuded	89.16	98.53	89.19	1.11	1.00
9	Bangladesh 1999-00	109.98	−0.48	−1.76	3.74	Excuded	80.33	87.82	78.33	1.09	0.98
10	Bangladesh 2004	96.26	−0.45	−1.17	2.56	Included (99%)	72.43	77.51	69.66	1.07	0.96
11	Bangladesh 2007	75.21	−0.51	−1.05	2.33	Included (99%)	58.24	62.00	56.32	1.06	0.97
12	Bangladesh 2011	64.08	−0.53	−0.66	1.93	Included (99%)	50.38	53.50	49.12	1.06	0.98
13	Bangladesh 2014	54.65	−0.65	0.10	0.82	Included (90%)	44.74	46.52	42.70	1.04	0.95
14	Benin 1996	181.71	1.17	−7.50	6.42	Excuded	103.75	120.23	109.64	1.16	1.06
15	Benin 2001	159.23	1.18	−7.28	4.83	Excuded	93.38	106.73	97.85	1.14	1.05
16	Benin 2006	134.23	1.21	−7.23	5.91	Excuded	75.84	91.26	83.84	1.20	1.11
17	Benin 2017-18	100.93	1.04	−6.45	5.91	Excuded	59.86	71.37	65.02	1.19	1.09
18	Bolivia 1989	137.93	0.38	0.17	−1.40	Included (90%)	89.85	100.32	94.16	1.12	1.05
19	Bolivia 1994	130.17	0.45	−0.95	0.26	Included (90%)	85.41	94.67	89.35	1.11	1.05
20	Bolivia 1998	98.45	0.14	0.44	−1.23	Included (90%)	73.44	75.29	70.16	1.03	0.96
21	Bolivia 2003	91.51	0.25	0.15	−1.14	Included (90%)	66.96	69.73	65.77	1.04	0.98
22	Bolivia 2008	76.50	0.10	0.15	−0.40	Included (90%)	58.02	59.83	56.34	1.03	0.97
23	Brazil 1986	96.04	0.04	0.12	−2.47	Excuded	82.09	74.20	76.84	0.90	0.94
24	Brazil 1996	55.44	0.32	1.20	−3.23	Excuded	47.43	43.43	46.88	0.92	0.99
25	Burkina Faso 1993	202.49	0.89	−5.66	5.25	Excuded	107.82	135.55	120.63	1.26	1.12
26	Burkina Faso 1998-99	220.18	1.45	−6.04	6.22	Excuded	107.29	139.19	129.99	1.30	1.21
27	Burkina Faso 2003	187.97	1.72	−6.46	4.99	Excuded	89.89	118.32	112.74	1.32	1.25
28	Burkina Faso 2010	147.28	1.66	−6.88	4.76	Excuded	77.41	95.48	90.94	1.23	1.17
29	Burundi 1987	180.28	1.54	−7.60	9.14	Excuded	86.37	115.72	108.46	1.34	1.26
30	Burundi 2010	126.75	0.95	−5.15	5.47	Excuded	78.78	88.52	79.85	1.12	1.01
31	Burundi 2016-17	81.00	1.23	−3.50	4.31	Excuded	49.43	57.34	53.75	1.16	1.09
32	Cambodia 2000	118.94	0.04	−0.63	2.50	Included (99%)	90.81	90.25	86.23	0.99	0.95
33	Cambodia 2005	105.84	−0.37	1.96	0.04	Included (99%)	88.27	83.98	79.31	0.95	0.90
34	Cambodia 2010	68.84	−0.44	0.31	0.99	Included (90%)	58.51	56.75	55.81	0.97	0.95
35	Cambodia 2014	47.88	−0.04	0.30	1.67	Included (99%)	38.60	39.00	40.47	1.01	1.05
36	Chad 1996-97	197.68	1.06	−6.26	5.33	Excuded	108.29	130.83	118.07	1.21	1.09
37	Chad 2004	198.61	1.02	−6.14	4.81	Excuded	113.90	131.74	118.10	1.16	1.04
38	Chad 2014-15	146.46	1.40	−6.46	4.48	Excuded	81.54	97.19	90.39	1.19	1.11
39	Colombia 1986	49.33	0.52	−0.98	−1.62	Included (99%)	38.93	38.25	39.24	0.98	1.01
40	Colombia 1990	34.78	0.86	−2.57	−1.36	Excuded	26.83	26.62	28.99	0.99	1.08
41	Colombia 1995	37.11	−0.18	0.65	−1.86	Included (99%)	30.91	30.93	30.41	1.00	0.98
42	Colombia 2000	27.71	−0.36	0.63	−1.13	Included (90%)	24.35	23.68	23.24	0.97	0.95
43	Colombia 2005	27.01	−0.01	0.35	−0.67	Included (90%)	23.15	22.42	22.70	0.97	0.98
44	Colombia 2010	22.19	0.06	0.98	−1.73	Included (99%)	18.73	18.40	18.90	0.98	1.01
45	Colombia 2015	18.72	0.18	1.08	−1.91	Included (99%)	15.83	15.39	16.03	0.97	1.01
46	Comoros 1996	107.88	0.04	−2.82	1.45	Excuded	80.67	82.55	77.80	1.02	0.96
47	Congo Democratic Republic 2007	121.59	1.06	−5.69	3.45	Excuded	93.00	104.77	103.94	1.13	1.12
48	Congo Democratic Republic 2013-14	80.40	1.47	−6.66	4.83	Excuded	64.42	74.23	81.13	1.15	1.26
49	Congo 2005	154.25	0.87	−4.00	3.04	Excuded	79.87	85.84	76.64	1.07	0.96
50	Congo 2011-12	109.65	1.42	−5.65	4.43	Excuded	46.72	56.02	53.53	1.20	1.15
51	Dominican Republic 1986	90.08	−0.14	−1.48	0.29	Included (99%)	69.72	71.04	66.32	1.02	0.95
52	Dominican Republic 1991	61.38	0.61	−1.31	−1.02	Included (90%)	44.30	46.67	47.23	1.05	1.07
53	Dominican Republic 1996	59.63	0.24	−1.07	−1.77	Included (99%)	47.38	46.85	46.65	0.99	0.98
54	Dominican Republic 2002	42.66	−0.01	−0.45	−1.07	Included (90%)	34.09	34.84	34.34	1.02	1.01
55	Dominican Republic 2007	36.49	−0.25	−0.14	−1.19	Included (90%)	32.02	30.62	30.13	0.96	0.94
56	Dominican Republic 2013	33.55	−0.79	2.05	−2.20	Excuded	30.27	29.55	27.68	0.98	0.91
57	Ecuador 1987	88.60	0.21	−0.43	−0.14	Included (90%)	62.86	67.94	64.49	1.08	1.03
58	Egypt 1988	130.46	0.15	−1.57	−0.79	Included (99%)	93.30	97.27	91.41	1.04	0.98
59	Egypt 1992	107.21	0.10	−1.48	−0.03	Included (99%)	79.62	81.66	76.75	1.03	0.96
60	Egypt 1995	95.02	0.15	−1.34	−0.67	Included (90%)	72.16	72.82	68.79	1.01	0.95
61	Egypt 2000	67.74	0.13	0.04	−1.15	Included (90%)	53.91	53.29	51.43	0.99	0.95
62	Egypt 2003	54.42	0.12	0.27	−1.97	Included (99%)	44.28	43.42	42.54	0.98	0.96
63	Egypt 2005	49.50	0.14	−0.09	−0.98	Included (90%)	39.94	39.63	39.32	0.99	0.98
64	Egypt 2008	33.02	0.05	0.56	−1.40	Included (90%)	28.36	27.11	27.37	0.96	0.97
65	Egypt 2014	30.29	−0.10	1.24	−0.99	Included (99%)	25.93	25.26	25.24	0.97	0.97
66	Ethiopia 2000	184.77	0.27	−5.22	6.90	Excuded	112.03	131.66	111.17	1.18	0.99
67	Ethiopia 2005	130.24	0.56	−4.59	6.83	Excuded	80.48	93.83	81.85	1.17	1.02
68	Ethiopia 2011	109.19	0.12	−3.77	5.32	Excuded	73.68	82.89	69.41	1.12	0.94
69	Ethiopia 2016	81.41	−0.28	−1.53	2.88	Included (99%)	61.51	65.44	53.98	1.06	0.88
70	Ghana 1988	149.87	0.73	−6.40	7.22	Excuded	80.68	105.00	92.35	1.30	1.14
71	Ghana 1993	131.53	0.62	−6.38	7.42	Excuded	74.24	94.15	82.04	1.27	1.11
72	Ghana 1998	107.90	1.34	−6.81	5.82	Excuded	59.88	73.97	69.03	1.24	1.15
73	Ghana 2003	105.74	0.48	−6.25	6.20	Excuded	63.78	78.07	68.27	1.22	1.07
74	Ghana 2008	83.57	0.65	−5.16	4.82	Excuded	52.24	61.97	54.62	1.19	1.05
75	Ghana 2014	69.43	0.31	−4.39	5.11	Excuded	47.22	53.71	47.17	1.14	1.00
76	Guatemala 1987	120.81	0.76	−3.12	1.09	Excuded	79.66	86.18	85.17	1.08	1.07
77	Guatemala 1995	78.52	0.51	−1.20	−0.64	Included (90%)	57.14	59.22	58.34	1.04	1.02
78	Guatemala 1998-99	63.60	0.59	−0.07	−0.96	Included (90%)	48.50	48.32	48.82	1.00	1.01
79	Guatemala 2014-15	38.04	0.27	1.54	−1.76	Included (99%)	29.80	30.48	31.00	1.02	1.04

	Survey	U5MR	k	Mean Prediction Error (%)		Selection	IMR			Ratio	
				0 to 8 mos	24 to 60 mos		Obs. (1)	k-Model (2)	MLT (3)	(2)/(1)	(3)/(1)
80	Guinea 1999	190.30	0.63	−6.03	6.00	Excuded	105.53	131.07	114.14	1.24	1.08
81	Guinea 2005	184.21	0.48	−6.10	4.71	Excuded	109.37	128.96	110.83	1.18	1.01
82	Guinea 2012	132.05	0.87	−5.75	5.15	Excuded	76.41	92.53	82.41	1.21	1.08
83	Guinea 2018	105.41	1.00	−5.39	4.37	Excuded	63.30	74.54	67.54	1.18	1.07
84	Guyana 2009	36.41	−0.37	−1.02	−0.19	Included (90%)	32.71	30.87	31.75	0.94	0.97
85	Haiti 1994-95	137.97	0.98	−2.45	1.95	Included (99%)	87.13	95.36	96.00	1.09	1.10
86	Haiti 2000	136.85	0.81	−2.56	2.20	Excuded	89.92	96.02	95.47	1.07	1.06
87	Haiti 2005-06	101.36	0.95	−2.35	0.84	Included (99%)	69.56	72.21	72.83	1.04	1.05
88	Haiti 2012	90.26	0.64	−2.36	1.43	Included (99%)	62.78	66.64	66.00	1.06	1.05
89	Haiti 2016-17	82.11	0.50	−1.94	1.67	Included (99%)	57.76	61.79	60.58	1.07	1.05
90	Honduras 2005-06	37.80	0.36	−0.18	−0.81	Included (90%)	29.25	30.08	30.79	1.03	1.05
91	Honduras 2011-12	28.92	0.03	−0.15	−0.61	Included (90%)	23.46	23.90	24.18	1.02	1.03
92	India 1992-93	118.53	−0.34	−2.36	3.05	Included (99%)	86.46	92.90	83.97	1.07	0.97
93	India 1998-99	99.81	−0.37	−2.28	3.11	Included (99%)	72.13	79.62	83.97	1.10	1.16
94	India 2005-06	85.47	−0.53	−1.74	2.62	Included (99%)	65.01	69.95	62.82	1.08	0.97
95	Indonesia 1987	108.86	0.63	−1.50	2.16	Included (99%)	74.44	79.22	77.77	1.06	1.04
96	Indonesia 1991	106.08	0.57	−1.49	2.14	Included (99%)	73.68	77.73	76.12	1.05	1.03
97	Indonesia 1994	92.03	0.42	−1.25	1.52	Included (90%)	66.10	69.13	66.80	1.05	1.01
98	Indonesia 1997	70.18	0.60	−0.88	1.31	Included (90%)	51.92	52.94	53.00	1.02	1.02
99	Indonesia 2002-03	53.23	0.33	−0.68	0.29	Included (90%)	42.21	41.78	41.98	0.99	0.99
100	Indonesia 2007	50.94	0.38	−1.31	1.00	Included (90%)	39.10	39.90	40.00	1.02	1.02
101	Indonesia 2012	42.37	0.20	−1.08	1.03	Included (90%)	33.05	33.99	34.01	1.03	1.03
102	Jordan 1990	41.83	−0.26	1.65	−2.19	Excuded	36.47	34.92	33.71	0.96	0.92
103	Jordan 1997	33.66	−0.21	1.79	−1.81	Included (99%)	28.90	28.23	27.79	0.98	0.96
104	Jordan 2002	28.75	−0.26	0.10	0.51	Included (90%)	24.01	24.33	24.05	1.01	1.00
105	Jordan 2007	22.62	−0.40	1.23	−1.19	Included (99%)	20.21	19.48	19.24	0.96	0.95
106	Jordan 2009	30.20	−0.35	0.42	0.27	Included (90%)	25.67	25.73	25.17	1.00	0.98
107	Jordan 2012	20.10	−0.22	0.34	−0.33	Included (90%)	17.14	17.09	17.18	1.00	1.00
108	Jordan 2017-18	16.63	0.10	−0.02	−1.66	Included (99%)	14.66	13.79	14.30	0.94	0.98
109	Kazakhstan 1995	47.87	0.67	1.80	−2.69	Excuded	40.81	36.71	40.97	0.90	1.00
110	Kazakhstan 1999	63.22	−0.15	0.01	−3.25	Excuded	55.33	51.14	52.99	0.92	0.96
111	Kyrgyz Republic 1997	75.44	−0.29	0.00	−1.89	Included (99%)	65.98	61.05	61.67	0.93	0.93
112	Kyrgyz Republic 2012	30.97	−0.15	−0.63	−0.14	Included (90%)	26.35	25.91	28.67	0.98	1.09
113	Liberia 1986	225.64	−0.20	−3.84	1.87	Excuded	149.12	163.66	151.71	1.10	1.02
114	Liberia 2007	138.12	1.00	−3.87	0.89	Excuded	91.28	95.33	97.09	1.04	1.06
115	Liberia 2013	111.90	0.96	−4.55	2.92	Excuded	70.15	79.01	79.27	1.13	1.13
116	Malawi 1992	237.66	1.12	−6.56	3.49	Excuded	135.87	153.13	140.77	1.13	1.04
117	Malawi 2000	200.04	1.17	−5.71	2.82	Excuded	111.47	131.00	118.90	1.18	1.07
118	Malawi 2004	163.25	1.21	−5.26	3.18	Excuded	96.14	108.88	99.57	1.13	1.04
119	Malawi 2010	124.62	1.28	−5.24	3.95	Excuded	72.53	84.81	78.22	1.17	1.08
120	Malawi 2015-16	74.30	0.92	−4.22	3.86	Excuded	46.26	54.34	49.96	1.17	1.08
121	Maldives 2009	27.86	−0.17	−1.28	1.56	Included (90%)	23.28	23.43	23.37	1.01	1.00
122	Maldives 2016-17	19.94	−0.29	−0.05	0.41	Included (90%)	17.01	17.05	17.05	1.00	1.00
123	Mali 1987	272.34	0.50	−7.96	9.22	Excuded	130.64	181.90	162.64	1.39	1.24
124	Mali 1995-96	249.44	0.45	−7.68	7.44	Excuded	133.65	169.13	147.43	1.27	1.10
125	Mali 2001	234.05	0.36	−6.76	6.80	Excuded	124.38	161.13	138.92	1.30	1.12
126	Mali 2006	213.25	0.72	−7.43	6.14	Excuded	112.98	143.91	127.16	1.27	1.13
127	Mali 2012-13	104.91	0.63	−5.03	6.91	Excuded	62.42	76.54	66.96	1.23	1.07
128	Mali 2018	111.67	1.14	−7.08	7.68	Excuded	60.31	77.62	71.06	1.29	1.18
129	Moldova 2005	26.14	−0.04	−1.03	1.64	Included (99%)	20.95	21.79	22.03	1.04	1.05
130	Morocco 1987	115.03	0.14	−0.78	−0.24	Included (90%)	80.76	86.82	84.03	1.08	1.04
131	Morocco 1992	83.27	0.17	0.54	−0.79	Included (90%)	63.08	64.34	65.44	1.02	1.04
132	Morocco 2003-04	53.53	−0.26	1.55	−0.86	Included (99%)	43.88	44.13	44.72	1.01	1.02
133	Myanmar 2015-16	71.20	−0.45	1.24	0.94	Included (99%)	58.69	58.65	53.65	1.00	0.91
134	Nepal 1996	138.64	−0.16	−3.46	4.60	Excuded	92.53	105.49	96.35	1.14	1.04
135	Nepal 2001	108.12	−0.17	−2.16	3.64	Excuded	76.93	84.24	77.13	1.10	1.00
136	Nepal 2006	81.13	−0.31	−0.71	0.90	Included (90%)	61.82	65.40	59.96	1.06	0.97
137	Nepal 2011	62.94	−0.68	−0.22	0.73	Included (90%)	53.69	53.27	48.32	0.99	0.90
138	Nepal 2016	45.98	−0.55	0.76	0.89	Included (90%)	38.98	39.18	36.68	1.01	0.94
139	Nicaragua 1998	54.94	0.56	0.32	−1.98	Included (99%)	44.95	42.22	43.40	0.94	0.97
140	Nicaragua 2001	43.50	0.74	0.76	−2.15	Included (99%)	34.79	33.32	34.85	0.96	1.00
141	Niger 1992	320.47	1.88	−8.50	6.37	Excuded	133.78	186.54	191.09	1.39	1.43
142	Niger 1998	302.12	1.82	−8.34	5.50	Excuded	136.74	178.19	179.37	1.30	1.31
143	Niger 2006	215.55	1.82	−8.11	5.70	Excuded	94.55	132.40	127.68	1.40	1.35
144	Niger 2012	150.36	2.11	−8.41	7.79	Excuded	65.23	93.66	92.64	1.44	1.42
145	Nigeria 1990	186.97	1.39	−7.74	6.25	Excuded	90.90	121.04	112.20	1.33	1.23
146	Nigeria 2003	211.10	1.04	−6.85	5.47	Excuded	107.72	138.88	126.69	1.29	1.18
147	Nigeria 2008	166.00	1.05	−6.55	5.21	Excuded	85.27	111.99	101.81	1.31	1.19
148	Nigeria 2013	142.92	1.12	−5.97	4.52	Excuded	76.53	97.34	88.23	1.27	1.15
149	Nigeria 2018	126.80	1.31	−7.41	6.81	Excuded	64.81	85.94	79.50	1.33	1.23
150	Pakistan 1990-91	118.85	−0.60	−0.10	0.03	Included (90%)	93.26	95.24	91.92	1.02	0.99
151	Pakistan 2006-07	90.54	−0.94	−0.78	1.25	Included (90%)	74.58	76.34	72.90	1.02	0.98
152	Pakistan 2012-13	97.02	−1.18	−0.73	1.04	Included (90%)	80.76	83.05	77.12	1.03	0.95
153	Pakistan 2017-18	76.67	−0.90	−0.30	0.79	Included (90%)	64.28	65.27	62.57	1.02	0.97

	Survey	U5MR	k	Mean Prediction Error (%)		Selection	IMR			Ratio	
				0 to 8 mos	24 to 60 mos		Obs. (1)	k-Model (2)	MLT (3)	(2)/(1)	(3)/(1)
154	Paraguay 1990	46.96	0.64	−0.81	−1.32	Included (90%)	36.11	36.15	37.22	1.00	1.03
155	Peru 1986	114.16	0.62	−0.43	−0.44	Included (90%)	79.06	82.77	76.41	1.05	0.97
156	Peru 1991-92	90.49	0.75	−0.41	−1.85	Included (99%)	62.79	66.15	62.85	1.05	1.00
157	Peru 1996	67.58	0.45	−0.09	−0.78	Included (90%)	49.57	51.77	49.24	1.04	0.99
158	Peru 2000	60.99	0.66	−0.31	1.03	Included (90%)	43.79	46.17	44.92	1.05	1.03
159	Peru 2004-06	41.99	0.59	0.36	0.37	Included (90%)	31.61	32.61	32.04	1.03	1.01
160	Peru 2007-08	41.99	0.59	0.36	0.37	Included (90%)	31.61	32.61	32.04	1.03	1.01
161	Peru 2009	30.75	0.64	1.10	−1.23	Included (90%)	23.84	24.08	23.80	1.01	1.00
162	Peru 2010	27.71	0.61	0.36	0.14	Included (90%)	20.95	21.81	21.51	1.04	1.03
163	Peru 2011	26.42	0.76	2.08	−1.93	Excuded	20.46	20.56	20.51	1.00	1.00
164	Peru 2012	25.36	0.36	0.16	−0.54	Included (90%)	19.65	20.45	19.69	1.04	1.00
165	Philippines 1993	63.14	1.70	−4.26	3.13	Excuded	38.56	43.69	43.57	1.13	1.13
166	Philippines 1998	54.26	1.10	−3.34	2.81	Excuded	35.91	39.87	38.64	1.11	1.08
167	Philippines 2003	40.42	0.70	−1.47	1.67	Included (99%)	29.64	31.16	30.48	1.05	1.03
168	Philippines 2008	37.01	0.56	−1.46	0.81	Included (99%)	27.90	28.99	28.26	1.04	1.01
169	Philippines 2013	31.70	0.82	−2.13	1.88	Included (99%)	23.22	24.42	24.77	1.05	1.07
170	Philippines 2017	27.23	0.26	−0.41	−0.09	Included (90%)	21.78	22.10	21.60	1.01	0.99
171	Sao Tome and Principe 2008-09	68.59	1.44	−3.25	4.22	Excuded	46.51	48.25	46.70	1.04	1.00
172	Senegal 1986	204.81	1.50	−7.76	8.12	Excuded	88.59	130.05	121.72	1.47	1.37
173	Senegal 1992-93	154.25	1.38	−6.94	8.21	Excuded	74.84	101.99	94.18	1.36	1.26
174	Senegal 1997	136.97	1.23	−5.98	7.01	Excuded	68.45	92.76	85.08	1.36	1.24
175	Senegal 2005	133.89	0.99	−6.72	7.11	Excuded	71.16	92.74	84.15	1.30	1.18
176	Senegal 2010-11	88.28	0.64	−4.84	6.25	Excuded	53.60	65.25	57.73	1.22	1.08
177	Senegal 2012-13	73.69	0.59	−3.43	5.88	Excuded	48.34	55.43	49.61	1.15	1.03
178	Senegal 2014	62.62	1.20	−4.03	4.64	Excuded	38.87	45.22	43.36	1.16	1.12
179	Senegal 2015	67.97	0.85	−4.58	4.93	Excuded	44.01	50.31	46.36	1.14	1.05
180	Senegal 2016	58.80	0.64	−3.12	3.74	Excuded	39.21	44.70	41.19	1.14	1.05
181	Senegal 2017	59.51	0.28	−2.55	2.99	Excuded	42.24	46.62	41.59	1.10	0.98
182	Sierra Leone 2013	170.27	0.85	−4.54	0.83	Excuded	108.10	116.50	112.56	1.08	1.04
183	Sri Lanka 1987	42.68	0.27	−1.49	2.91	Included (99%)	32.72	34.05	34.35	1.04	1.05
184	Sudan 1989-90	133.99	0.85	−5.68	3.78	Excuded	76.90	93.95	83.54	1.22	1.09
185	Tajikistan 2012	47.50	0.43	−0.54	−1.19	Included (90%)	38.31	37.18	40.97	0.97	1.07
186	Tajikistan 2017	32.40	0.55	−1.11	−1.36	Included (90%)	26.69	25.51	28.45	0.96	1.07
187	Thailand 1987	48.80	−0.13	0.44	1.01	Included (90%)	38.33	39.99	38.38	1.04	1.00
188	Timor-Leste 2009-10	80.71	0.78	−2.27	0.49	Included (99%)	57.12	59.39	65.36	1.04	1.14
189	Togo 1988	155.78	0.93	−7.54	8.43	Excuded	83.29	106.86	95.36	1.28	1.14
190	Togo 1998	141.73	1.00	−8.40	8.52	Excuded	79.97	97.55	88.20	1.22	1.10
191	Togo 2013-14	91.59	1.27	−6.49	5.38	Excuded	51.89	64.03	59.77	1.23	1.15
192	Trinidad and Tobago 1987	33.79	−0.68	−0.02	−0.67	Included (90%)	30.34	29.50	29.83	0.97	0.98
193	Tunisia 1988	72.91	0.31	−0.83	−0.46	Included (90%)	55.88	56.23	54.80	1.01	0.98
194	Turkey 1993	80.31	−0.29	0.45	−2.06	Included (99%)	68.46	64.67	59.26	0.94	0.87
195	Turkey 1998	59.33	−0.11	−0.32	−0.47	Included (90%)	48.35	48.03	46.00	0.99	0.95
196	Turkey 2003	46.50	0.20	0.71	−0.44	Included (90%)	37.85	37.15	37.10	0.98	0.98
197	Turkey 2008	32.64	0.66	−2.32	1.03	Included (99%)	25.10	25.47	27.09	1.01	1.08
198	Turkey 2013	19.31	0.42	0.91	−2.89	Excuded	17.00	15.56	16.66	0.92	0.98
199	Ukraine 2007	18.77	−0.60	2.74	−1.52	Excuded	16.60	16.49	16.08	0.99	0.97
200	Uzbekistan 1996	54.92	0.44	−0.80	−1.97	Included (99%)	43.32	42.63	46.45	0.98	1.07
201	Vietnam 1997	45.66	0.19	−2.47	3.35	Excuded	34.81	36.55	33.60	1.05	0.97
202	Vietnam 2002	33.42	0.16	−1.83	4.05	Excuded	24.97	27.18	25.92	1.09	1.04
203	Yemen 1991-92	124.78	0.34	−1.38	0.62	Included (99%)	89.14	91.90	94.31	1.03	1.06
204	Yemen 2013	55.28	−0.01	−0.15	−1.28	Included (90%)	45.44	44.56	44.20	0.98	0.97
