# Supplementary material for: Estimating the infant mortality rate from DHS birth histories in the presence of age heaping
Source: PLoS One. 2021 Nov 3;16(11):e0259304. doi: 10.1371/journal.pone.0259304 (PMC8565760; doi:10.1371/journal.pone.0259304)
Supplement: S2 Appendix — (PDF) [file pone.0259304.s002.pdf]

S2 Appendix : Graphical results for individual DHS surveys

The k-model was fitted to the observed q(x) values using ages 0-8 and 24-60 months only. Light green indicates a very good fit (Prediction errors within the 90% maximum values). Dark green indicates a relatively good fit (Prediction errors between the 90% and 99% maximum values). Red indicates a bad fit and exclusion from the analysis (Prediction errors outside the 99% maximum values). The ticker vertical line indicates the comparison between the observed and predicted infant mortality rate (IMR).

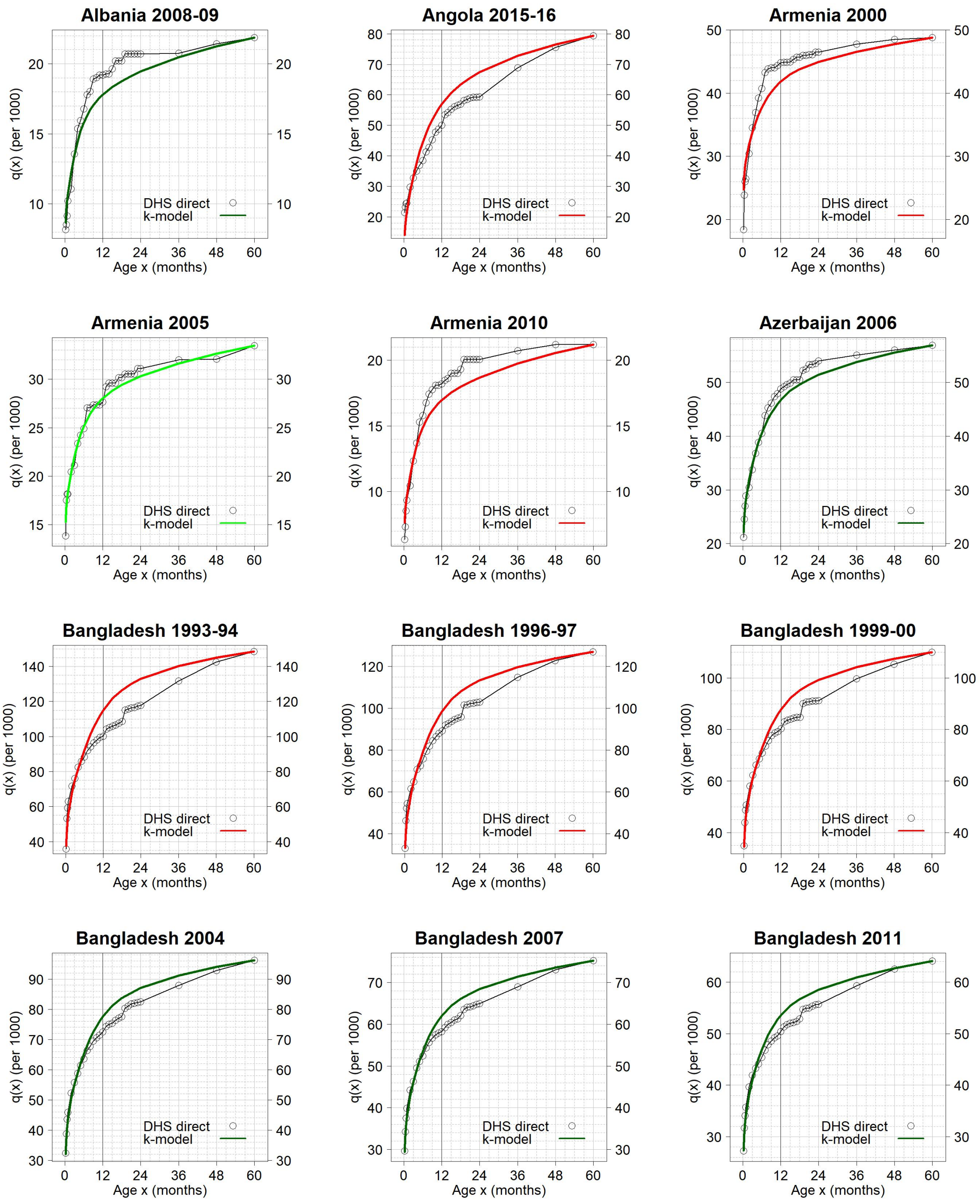
Albania 2008-09
Armenia 2005
Bangladesh 1993-94
Bangladesh 2004
Angola 2015-16
Armenia 2010
Bangladesh 1996-97
Bangladesh 2007
Armenia 2000
Azerbaijan 2006
Bangladesh 1999-00
Bangladesh 2011
q(x) (per 1000)
Age x (months)
DHS direct
k-model

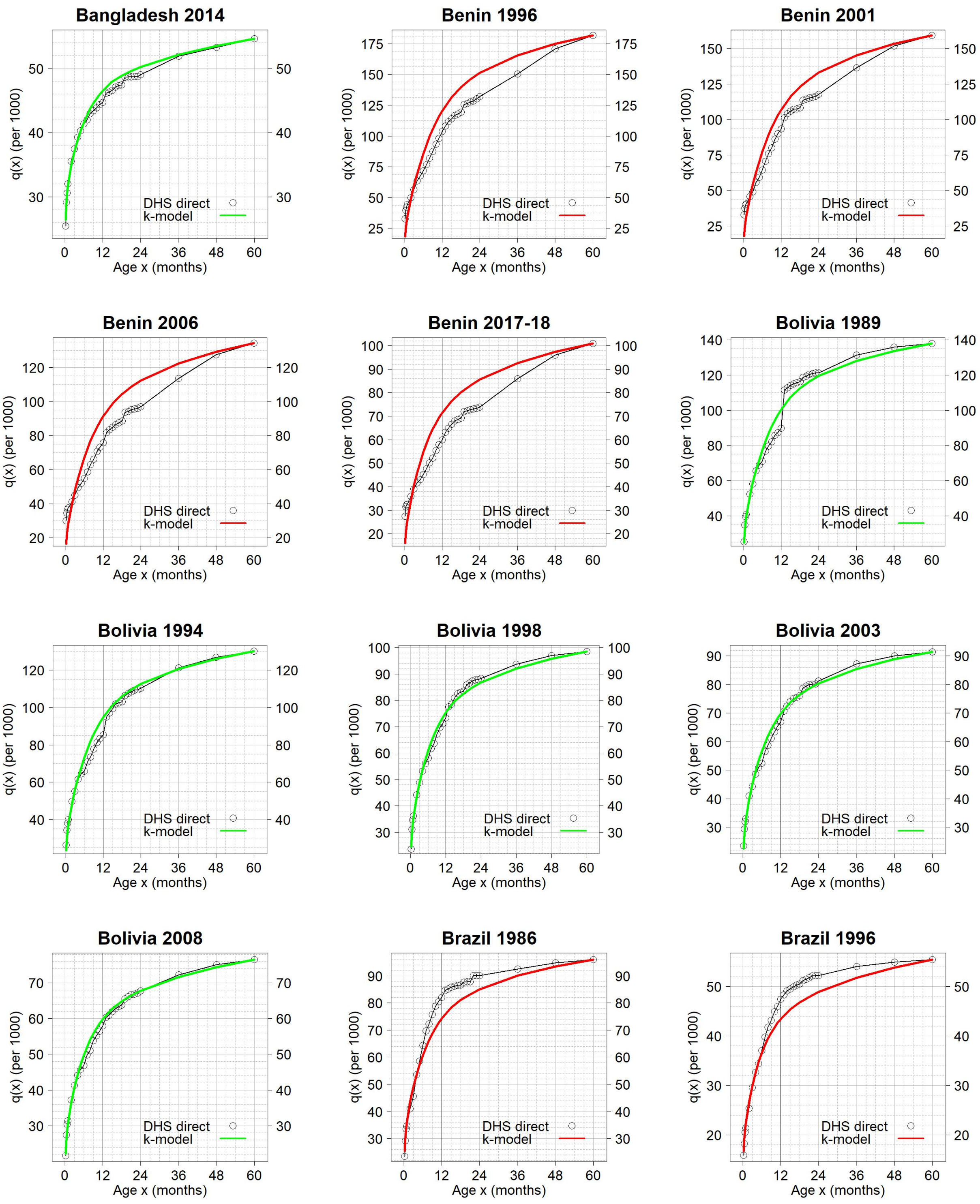
Bangladesh 2014
Benin 1996
Benin 2001
Benin 2006
Benin 2017-18
Bolivia 1989
Bolivia 1994
Bolivia 1998
Bolivia 2003
Bolivia 2008
Brazil 1986
Brazil 1996
q(x) (per 1000)
Age x (months)
DHS direct
k-model

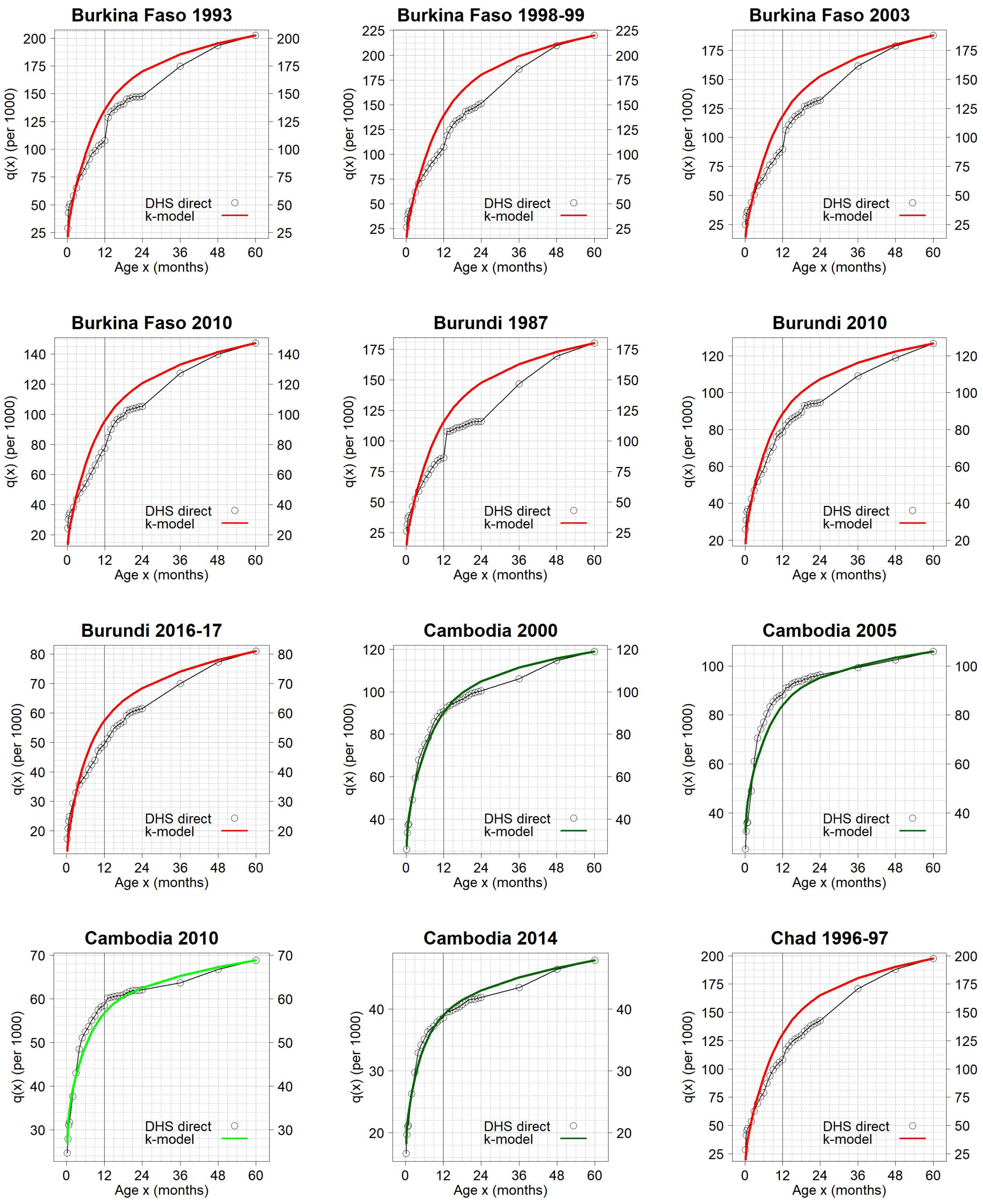
Burkina Faso 1993
Burkina Faso 1998-99
Burkina Faso 2003
Burkina Faso 2010
Burundi 1987
Burundi 2010
Burundi 2016-17
Cambodia 2000
Cambodia 2005
Cambodia 2010
Cambodia 2014
Chad 1996-97
q(x) (per 1000)
Age x (months)
DHS direct
k-model

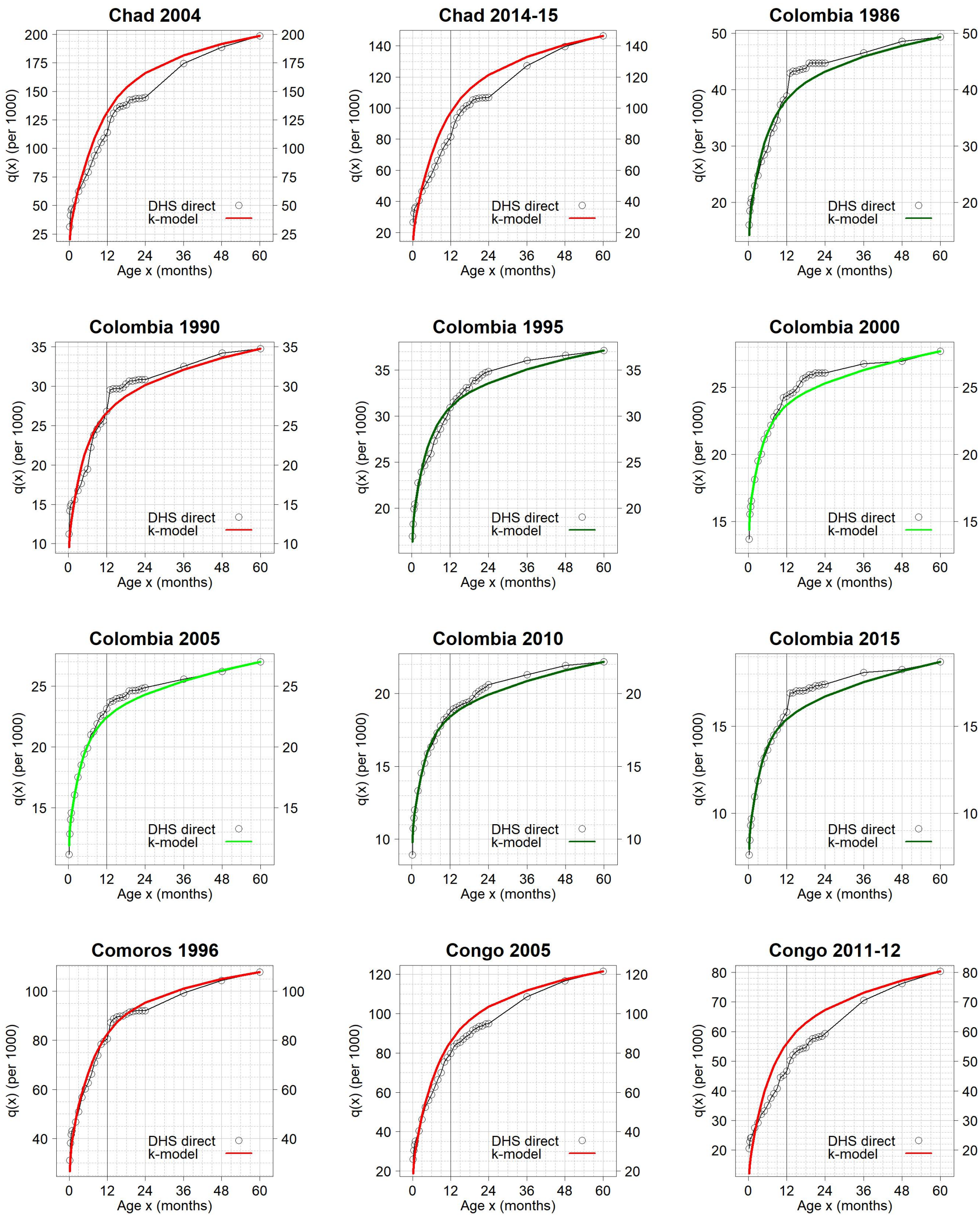
Chad 2004
Chad 2014-15
Colombia 1986
Colombia 1990
Colombia 1995
Colombia 2000
Colombia 2005
Colombia 2010
Colombia 2015
Comoros 1996
Congo 2005
Congo 2011-12
q(x) (per 1000)
Age x (months)
DHS direct
k-model

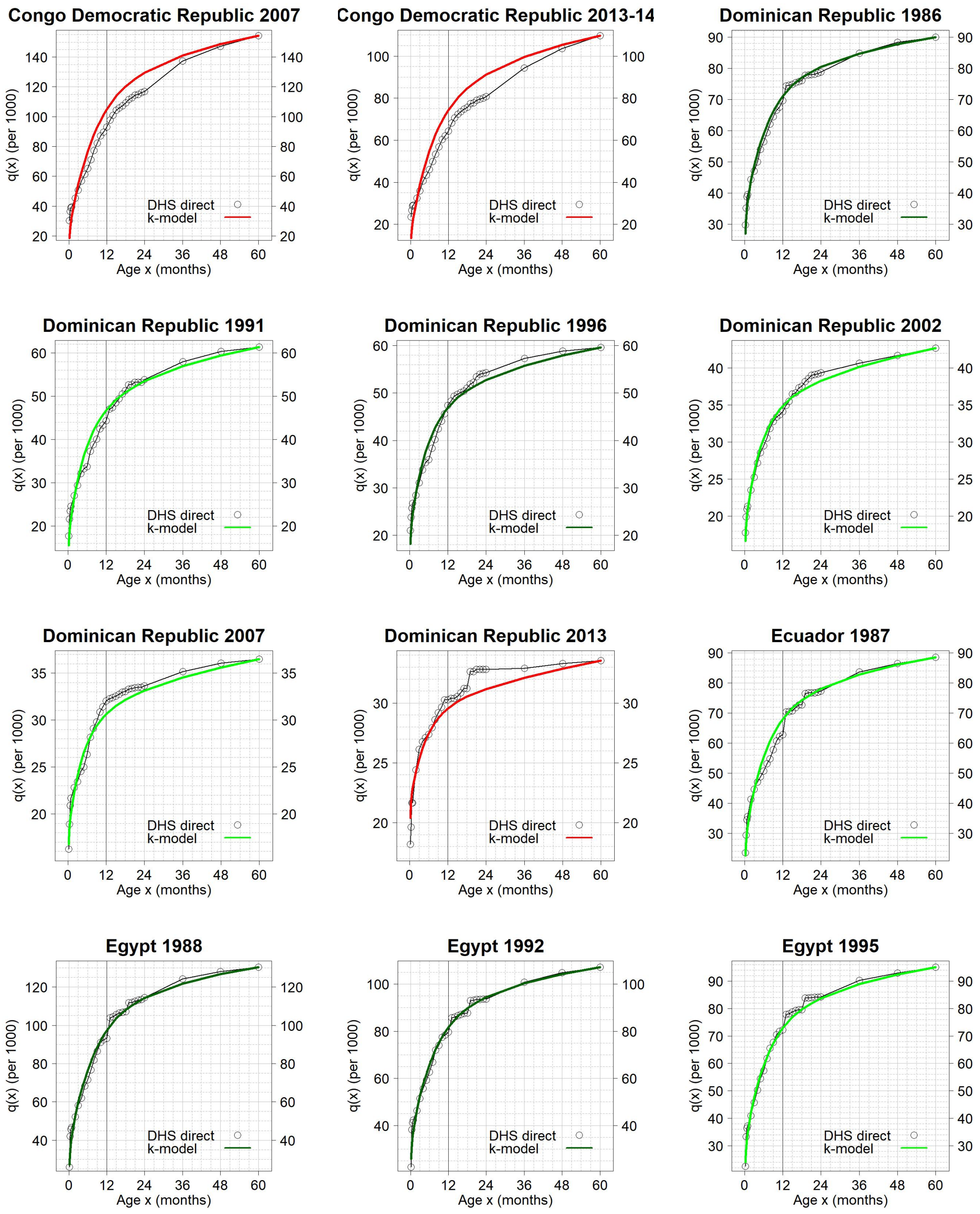
Congo Democratic Republic 2007
Congo Democratic Republic 2013-14
Dominican Republic 1986
Dominican Republic 1991
Dominican Republic 1996
Dominican Republic 2002
Dominican Republic 2007
Dominican Republic 2013
Ecuador 1987
Egypt 1988
Egypt 1992
Egypt 1995
q(x) (per 1000)
Age x (months)
DHS direct
k-model

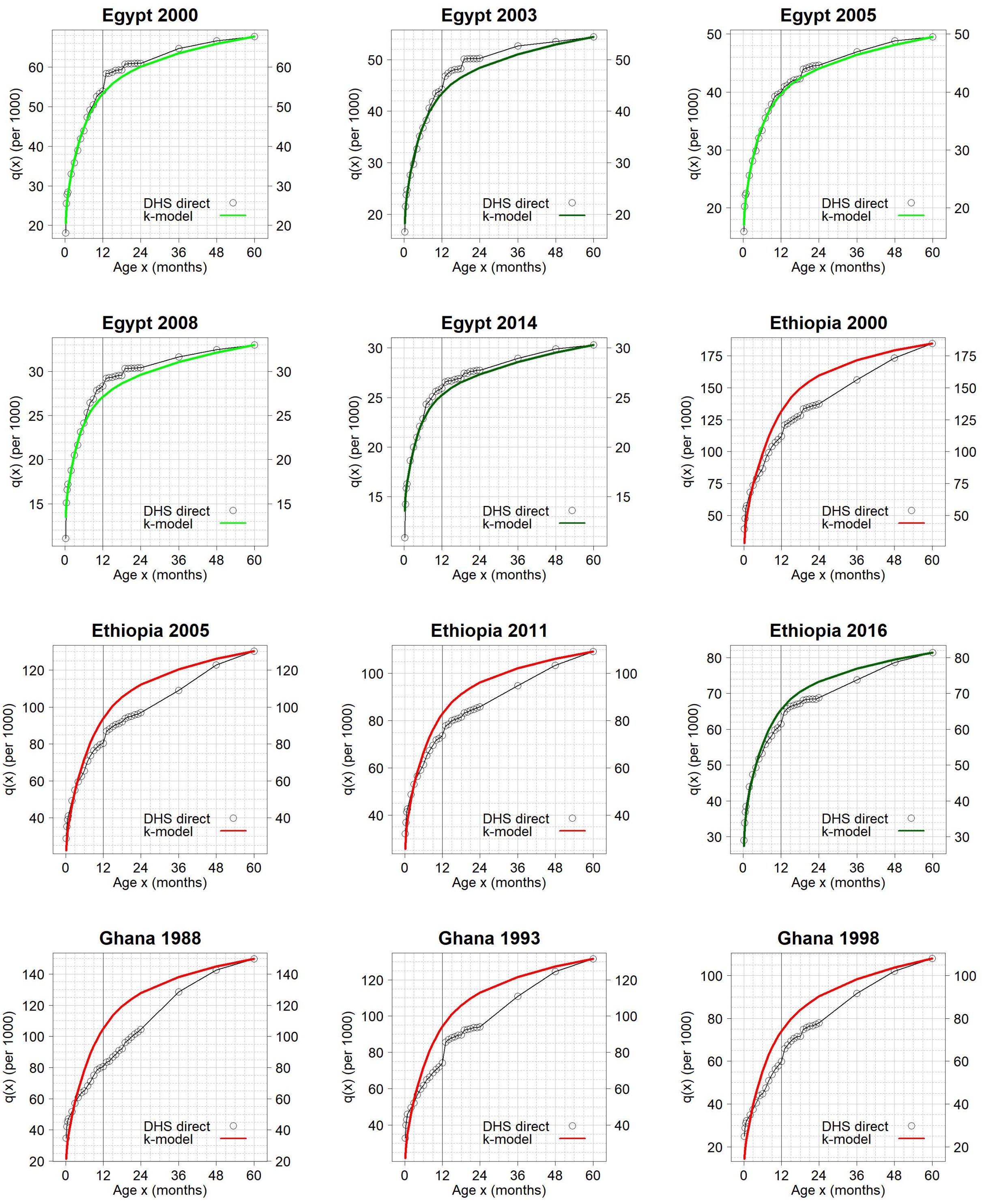
Egypt 2000
Egypt 2003
Egypt 2005
Egypt 2008
Egypt 2014
Ethiopia 2000
Ethiopia 2005
Ethiopia 2011
Ethiopia 2016
Ghana 1988
Ghana 1993
Ghana 1998
q(x) (per 1000)
Age x (months)
0 12 24 36 48 60
DHS direct
k-model

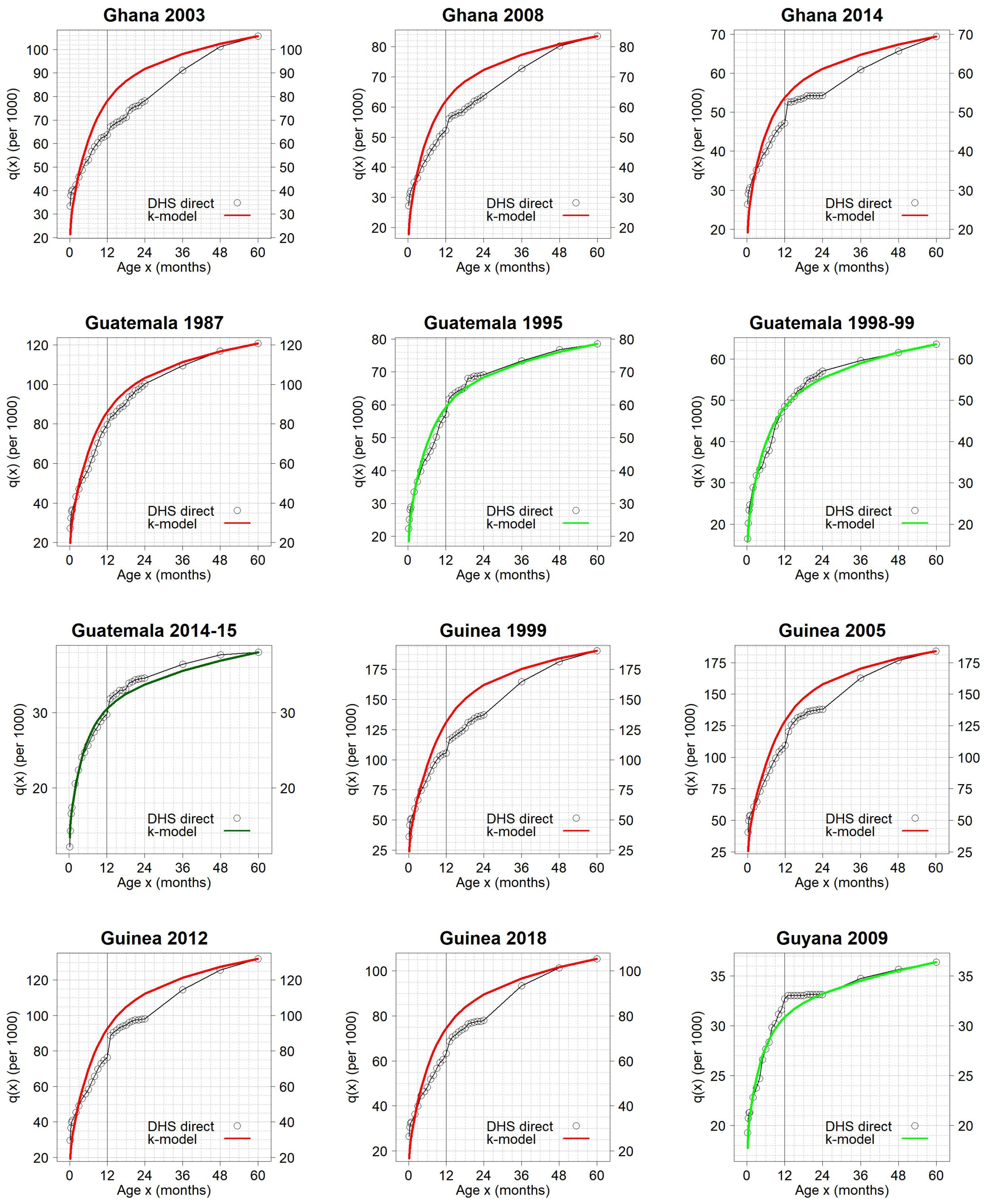
Ghana 2003
Ghana 2008
Ghana 2014
Guatemala 1987
Guatemala 1995
Guatemala 1998-99
Guatemala 2014-15
Guinea 1999
Guinea 2005
Guinea 2012
Guinea 2018
Guyana 2009
q(x) (per 1000)
Age x (months)
DHS direct
k-model

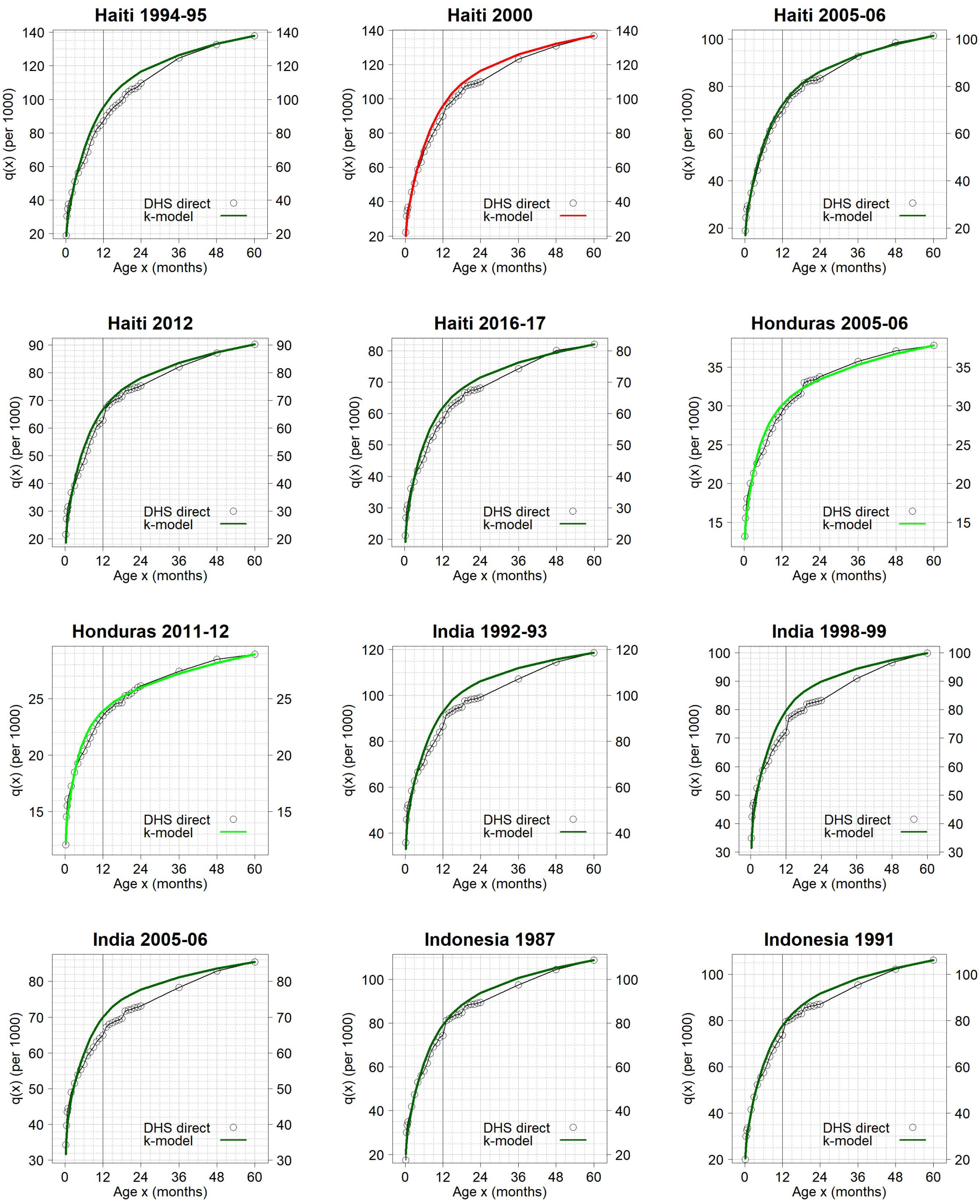
Haiti 1994-95
Haiti 2000
Haiti 2005-06
Haiti 2012
Haiti 2016-17
Honduras 2005-06
Honduras 2011-12
India 1992-93
India 1998-99
India 2005-06
Indonesia 1987
Indonesia 1991
q(x) (per 1000)
Age x (months)
DHS direct
k-model

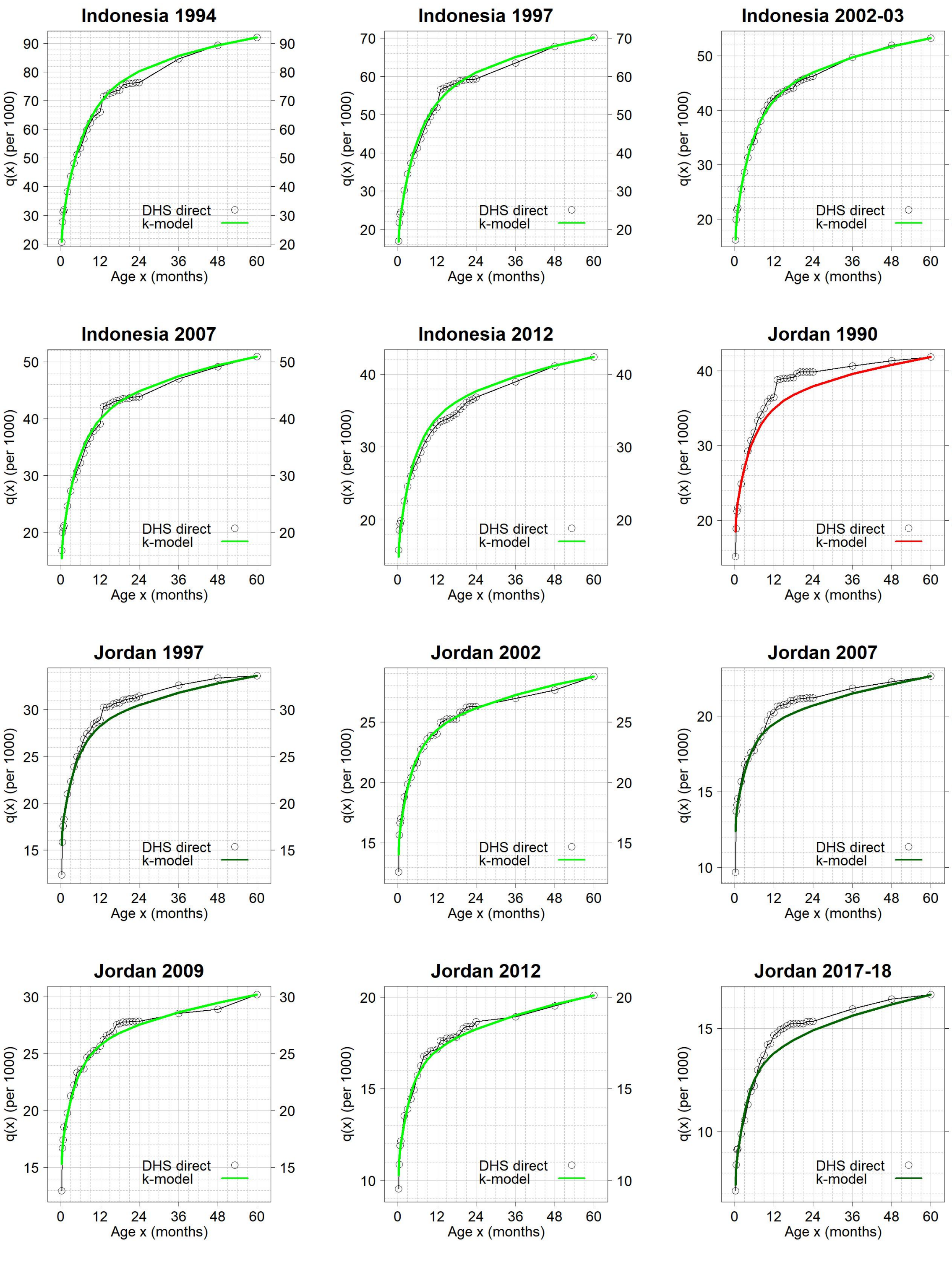
Indonesia 1994
Indonesia 1997
Indonesia 2002-03
Indonesia 2007
Indonesia 2012
Jordan 1990
Jordan 1997
Jordan 2002
Jordan 2007
Jordan 2009
Jordan 2012
Jordan 2017-18
q(x) (per 1000)
Age x (months)
DHS direct
k-model

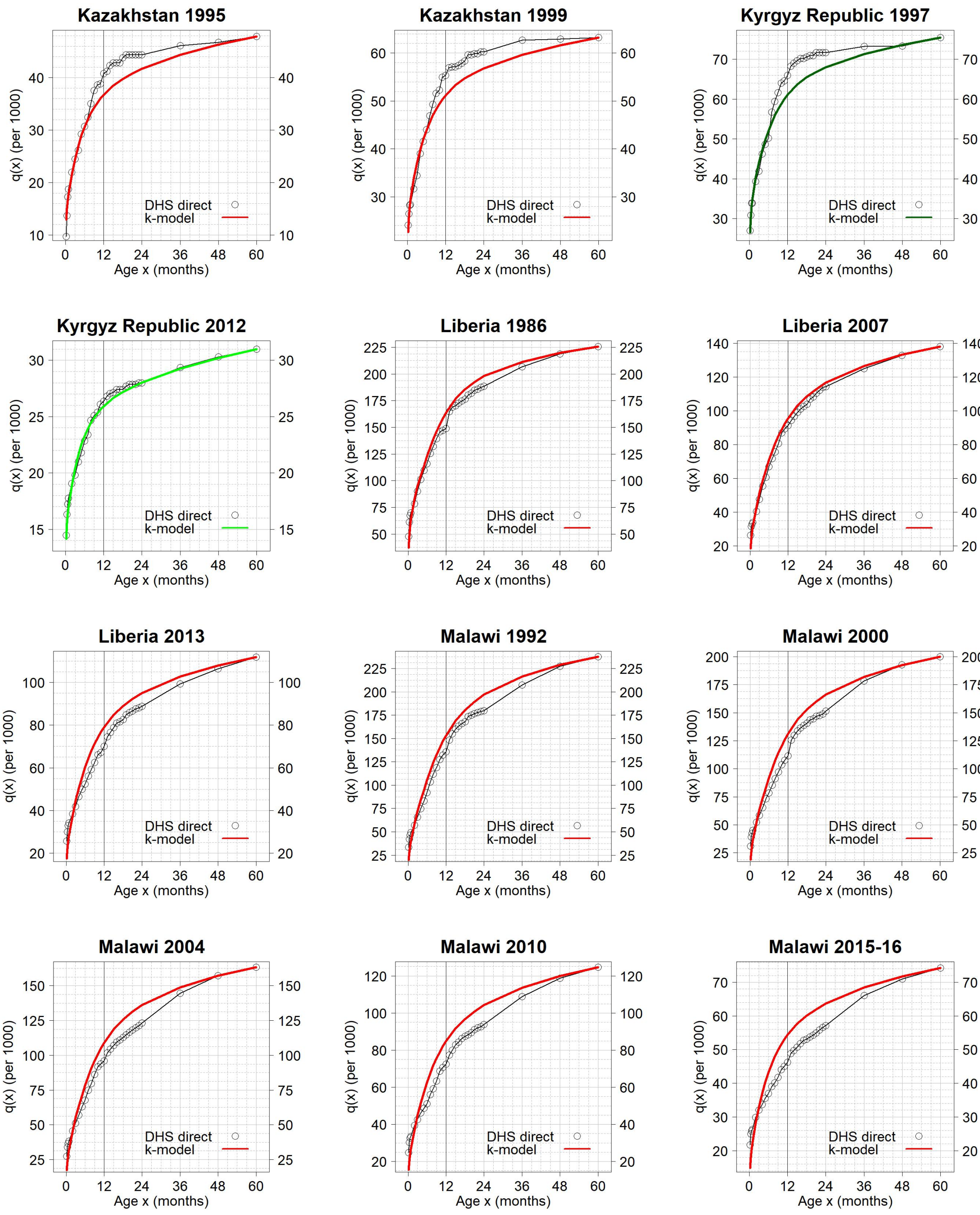
Kazakhstan 1995
Kazakhstan 1999
Kyrgyz Republic 1997
Kyrgyz Republic 2012
Liberia 1986
Liberia 2007
Liberia 2013
Malawi 1992
Malawi 2000
Malawi 2004
Malawi 2010
Malawi 2015-16
q(x) (per 1000)
Age x (months)
DHS direct
k-model

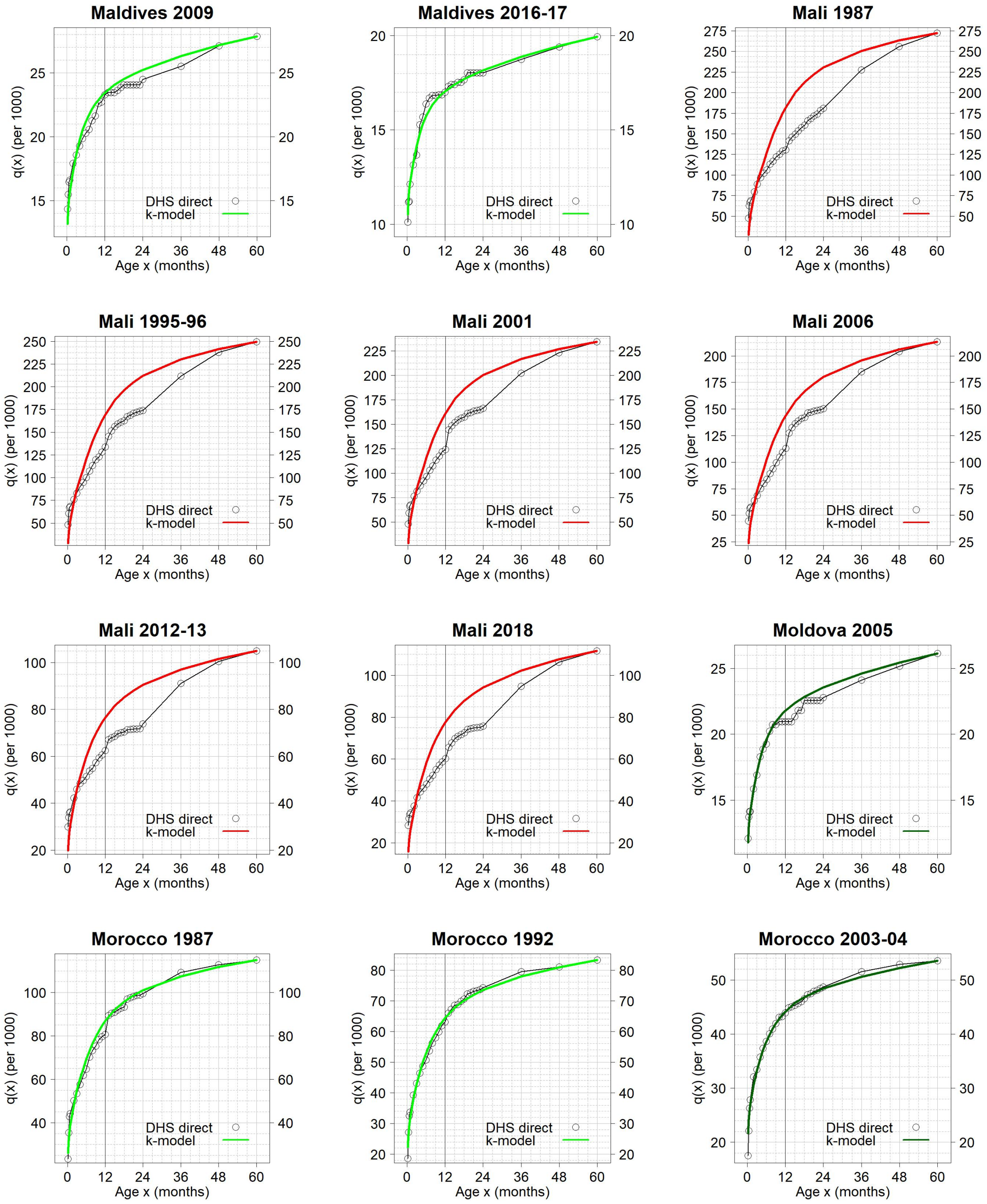
Maldives 2009
Maldives 2016-17
Mali 1987
Mali 1995-96
Mali 2001
Mali 2006
Mali 2012-13
Mali 2018
Moldova 2005
Morocco 1987
Morocco 1992
Morocco 2003-04
q(x) (per 1000)
Age x (months)
DHS direct
k-model

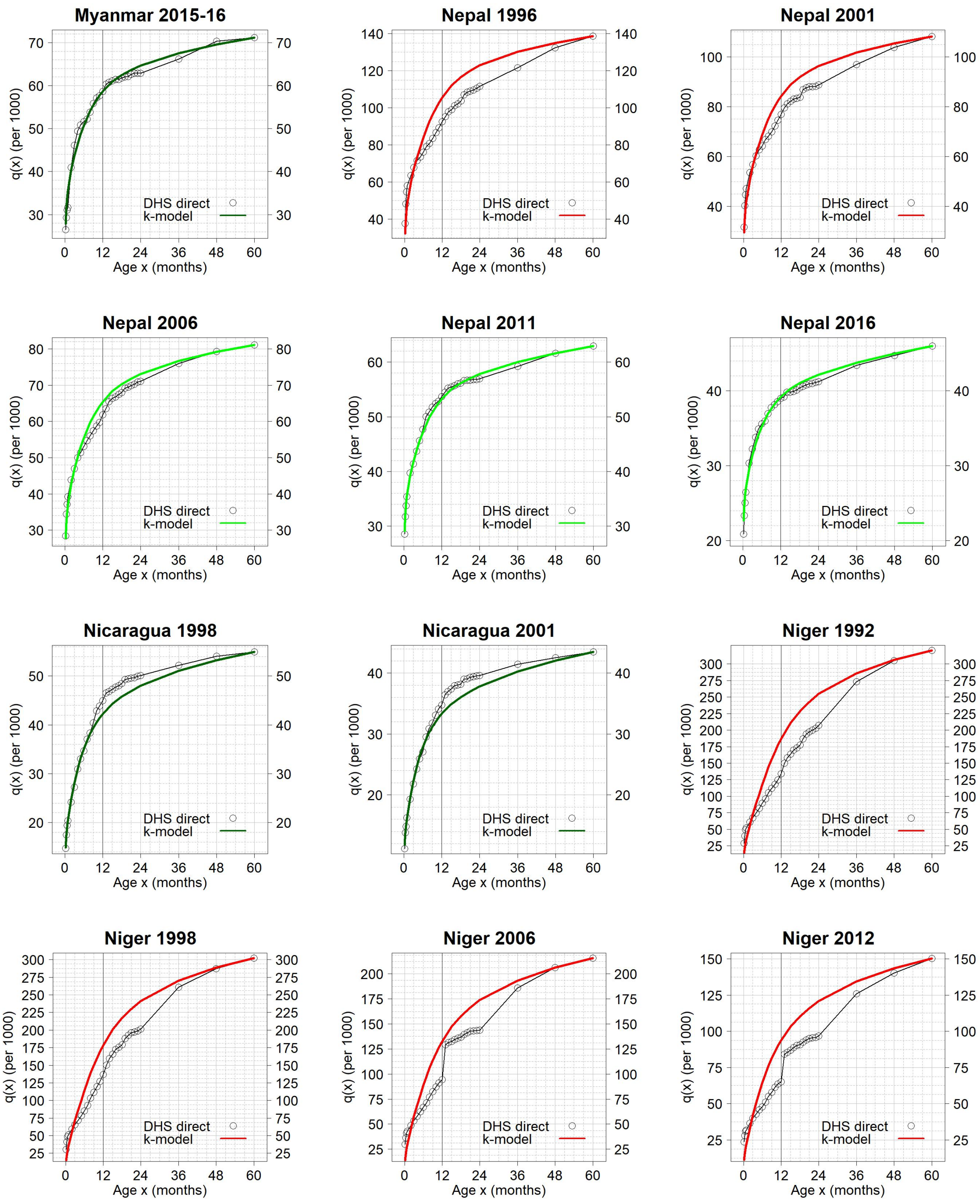
Myanmar 2015-16
Nepal 1996
Nepal 2001
Nepal 2006
Nepal 2011
Nepal 2016
Nicaragua 1998
Nicaragua 2001
Niger 1992
Niger 1998
Niger 2006
Niger 2012
q(x) (per 1000)
Age x (months)
DHS direct
k-model

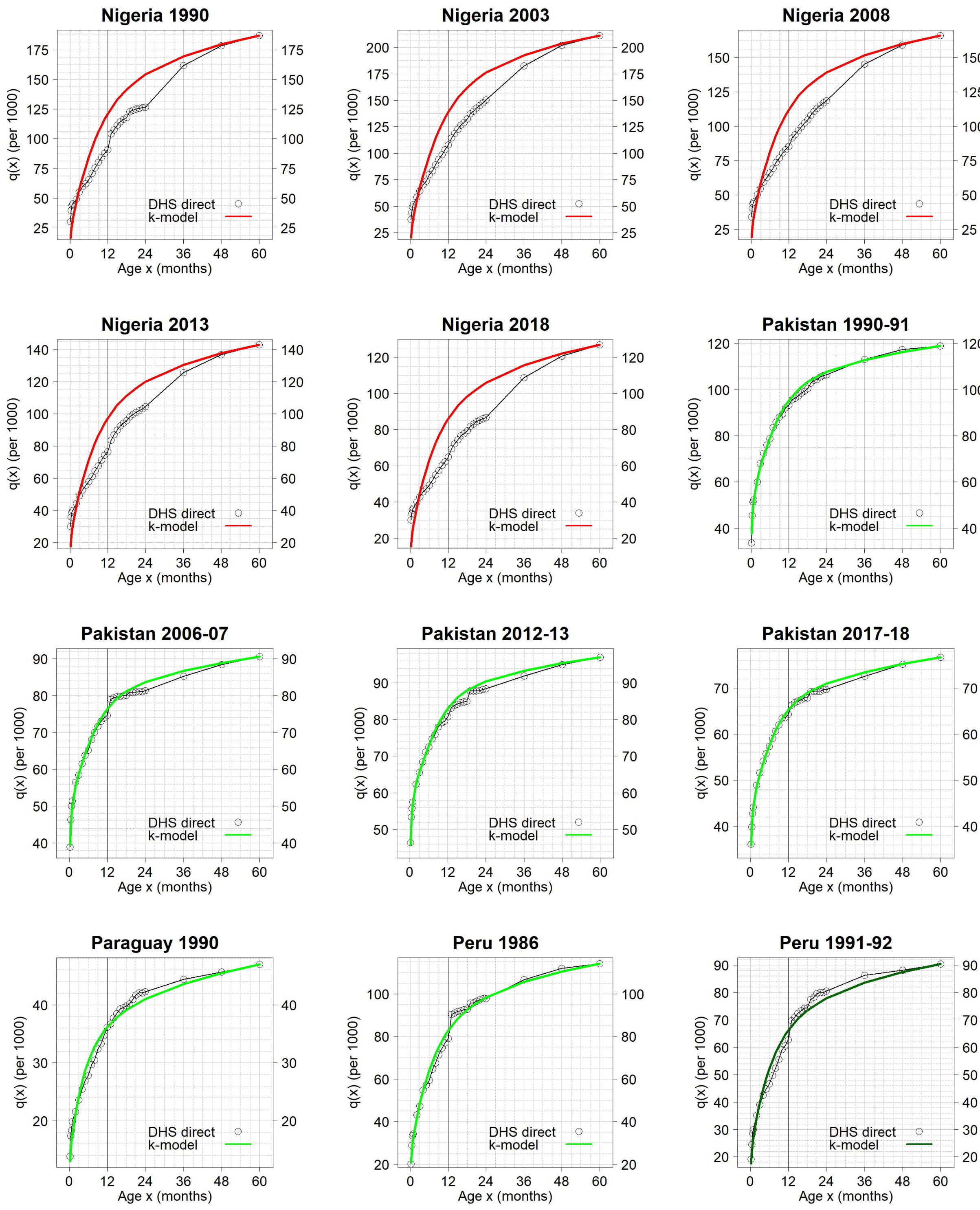
Nigeria 1990
Nigeria 2003
Nigeria 2008
Nigeria 2013
Nigeria 2018
Pakistan 1990-91
Pakistan 2006-07
Pakistan 2012-13
Pakistan 2017-18
Paraguay 1990
Peru 1986
Peru 1991-92
q(x) (per 1000)
Age x (months)
DHS direct
k-model

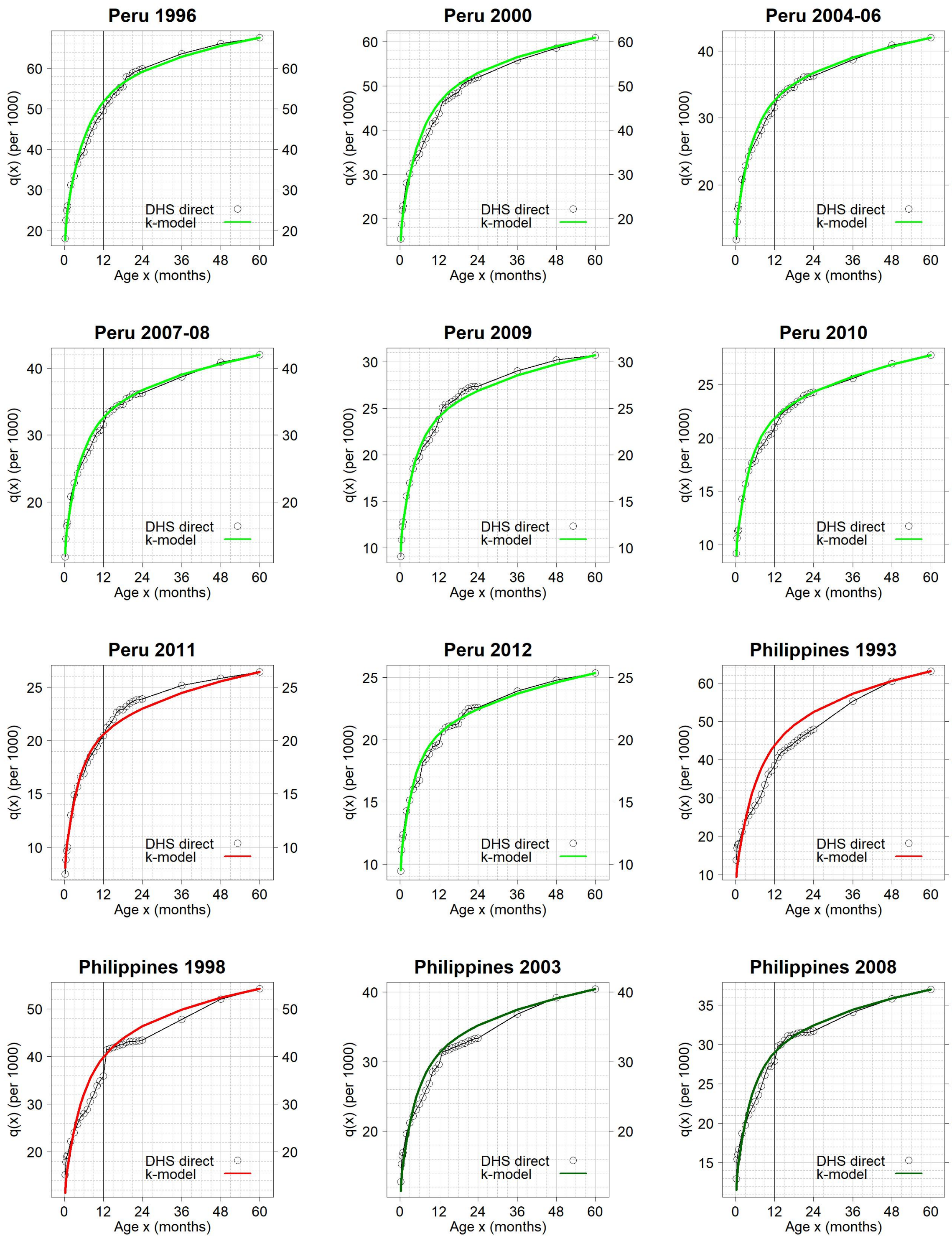
Peru 1996
Peru 2000
Peru 2004-06
Peru 2007-08
Peru 2009
Peru 2010
Peru 2011
Peru 2012
Philippines 1993
Philippines 1998
Philippines 2003
Philippines 2008
q(x) (per 1000)
Age x (months)
DHS direct
k-model

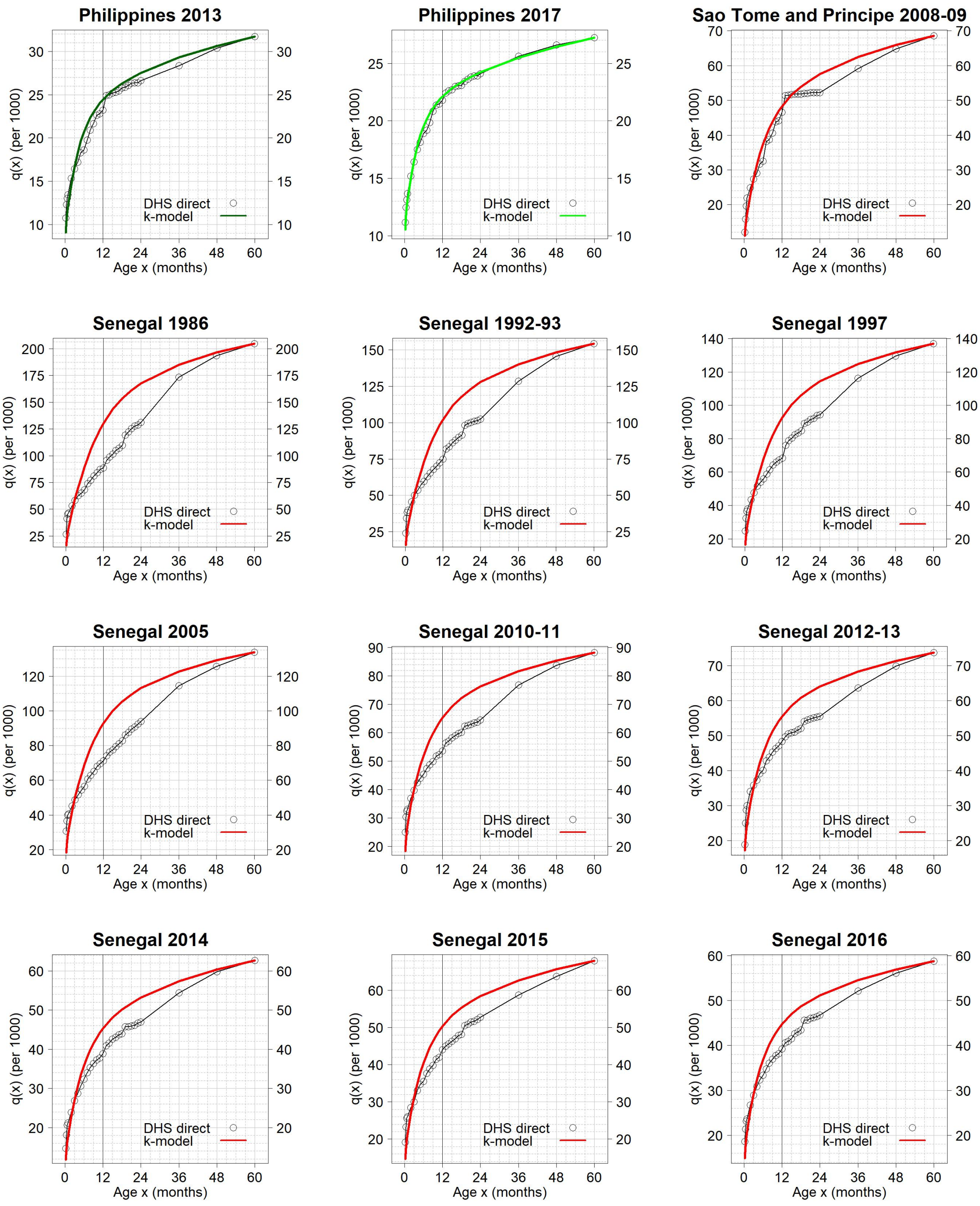
Philippines 2013
Philippines 2017
Sao Tome and Principe 2008-09
Senegal 1986
Senegal 1992-93
Senegal 1997
Senegal 2005
Senegal 2010-11
Senegal 2012-13
Senegal 2014
Senegal 2015
Senegal 2016
q(x) (per 1000)
Age x (months)
DHS direct
k-model

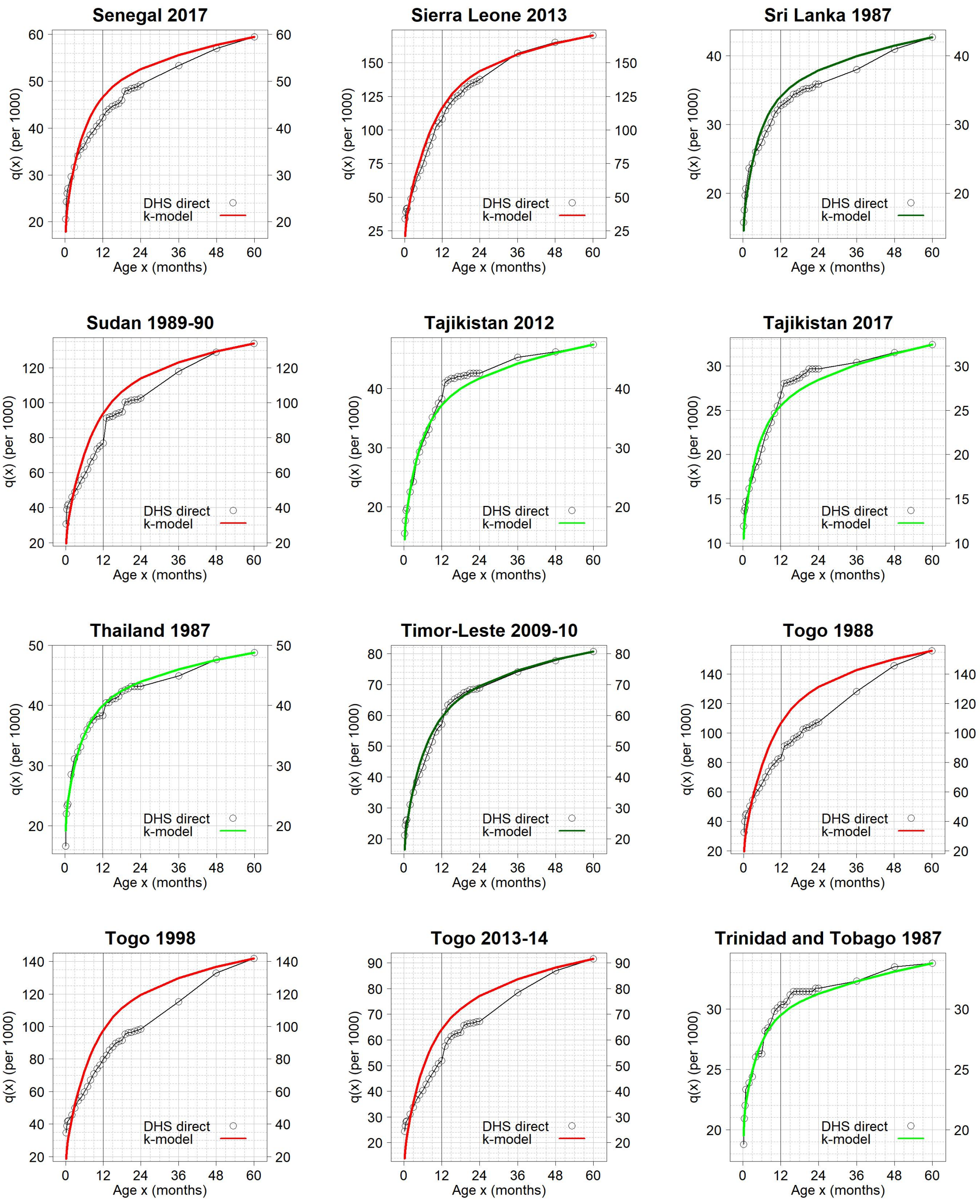

Senegal 2017
Sierra Leone 2013
Sri Lanka 1987
Sudan 1989-90
Tajikistan 2012
Tajikistan 2017
Thailand 1987
Timor-Leste 2009-10
Togo 1988
Togo 1998
Togo 2013-14
Trinidad and Tobago 1987
q(x) (per 1000)
Age x (months)
DHS direct
k-model

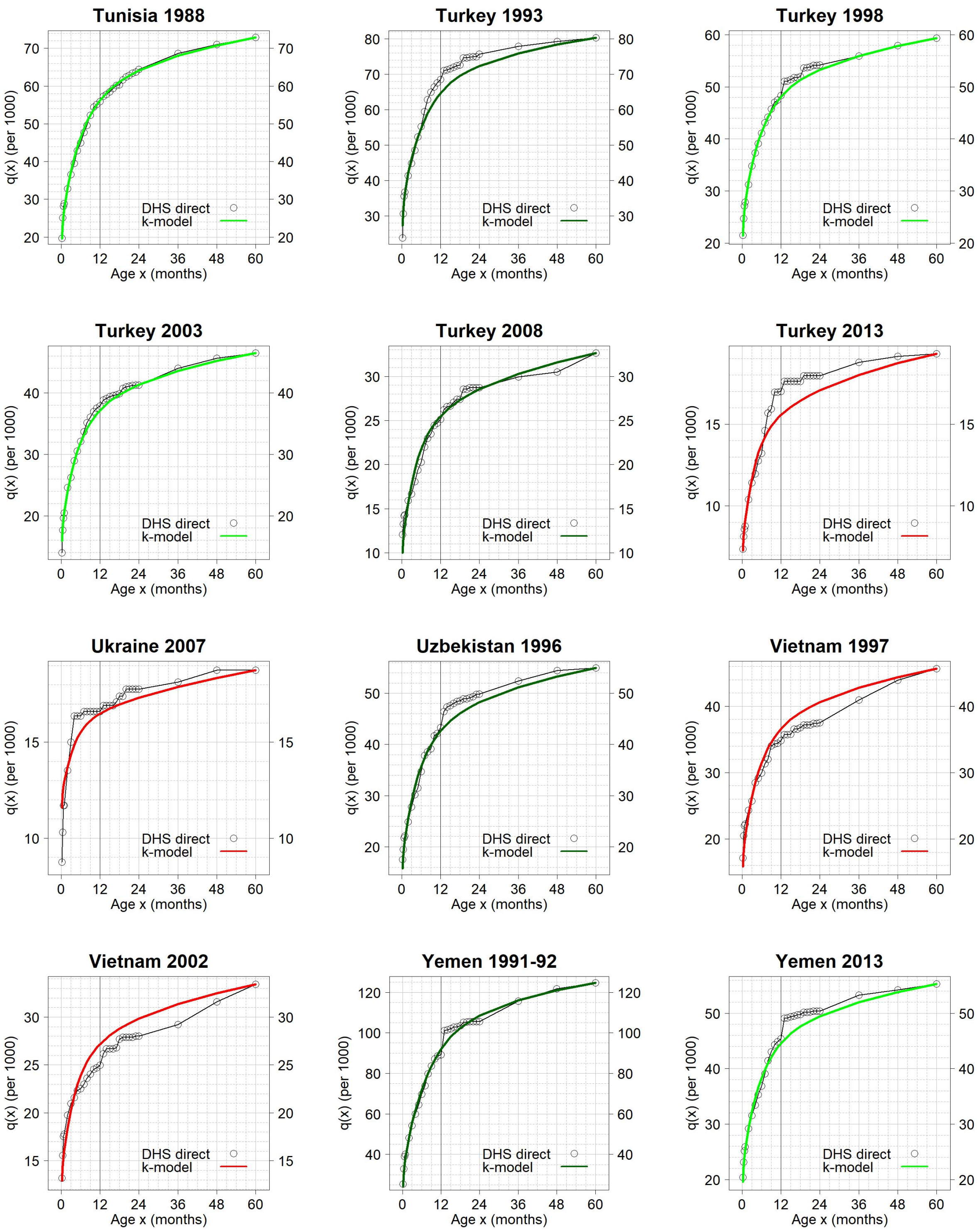
Tunisia 1988
Turkey 1993
Turkey 1998
Turkey 2003
Turkey 2008
Turkey 2013
Ukraine 2007
Uzbekistan 1996
Vietnam 1997
Vietnam 2002
Yemen 1991-92
Yemen 2013
q(x) (per 1000)
Age x (months)
DHS direct
k-model
